# A system dynamics-based scenario analysis of residential solid waste management in Kisumu, Kenya

**DOI:** 10.1016/j.scitotenv.2021.146200

**Published:** 2021-07-10

**Authors:** K. Dianati, L. Schäfer, J. Milner, A. Gómez-Sanabria, H. Gitau, J. Hale, H. Langmaack, G. Kiesewetter, K. Muindi, B. Mberu, N. Zimmermann, S. Michie, P. Wilkinson, M. Davies

**Affiliations:** a Institute for Environmental Design and Engineering (IEDE), Bartlett, UCL, UK; b Buro Happold, UK; c London School of Hygiene and Tropical Medicine (LSHTM), UK; d International Institute for Applied Systems Analysis (IIASA), Austria; e African Population and Health Research Centre (APHRC), Kenya; f UCL Centre for Behaviour Change (CBC), UK

**Keywords:** AD, anaerobic digestion, BC, black carbon, CO, carbon monoxide, COP, conference of the parties, DOC, degradable organic carbon, EU, European Union, GBD, Global Burden of Disease, GHG, greenhouse gas, GWP, global warming potential, HDI, human development index, ICS, improved cookstove, IHD, ischaemic heart disease, IPCC, Intergovernmental Panel on Climate Change, KISWAMP, Kisumu Integrated Solid Waste Management Plan, KNBS, Kenyan National Bureau of Statistics, LCA, life cycle assessment, LPG, liquefied petroleum gas, LRI, lower respiratory infections, MJ, megajoule, MSW, municipal solid waste, MSWM, municipal solid waste management, MW, megawatt, PM, particulate matter, SD, system dynamics, SDG, sustainable development goals, SSA, sub-Saharan Africa, SWM, solid waste management, WHO, World Health Organization, WtE, waste-to-energy, Municipal solid waste management, System dynamics, Greenhouse gas emissions, GHG accounting, Health impact assessment, Kisumu

## Abstract

The problem of solid waste management presents an issue of increasing importance in many low-income settings, including the progressively urbanised context of Kenya. Kisumu County is one such setting with an estimated 500 t of waste generated per day and with less than half of it regularly collected. The open burning and natural decay of solid waste is an important source of greenhouse gas (GHG) emissions and atmospheric pollutants with adverse health consequences. In this paper, we use system dynamics modelling to investigate the expected impact on GHG and PM_2.5_ emissions of (i) a waste-to-biogas initiative and (ii) a regulatory ban on the open burning of waste in landfill. We use life tables to estimate the impact on mortality of the reduction in PM_2.5_ exposure. Our results indicate that combining these two interventions can generate over 1.1 million tonnes of cumulative savings in GHG emissions by 2035, of which the largest contribution (42%) results from the biogas produced replacing unclean fuels in household cooking. Combining the two interventions is expected to reduce PM_2.5_ emissions from the waste and residential sectors by over 30% compared to our baseline scenario by 2035, resulting in at least around 1150 cumulative life years saved over 2021–2035. The contribution and novelty of this study lies in the quantification of a potential waste-to-biogas scenario and its environmental and health impact in Kisumu for the first time.

## Highlights

• We present a system dynamics study of solid waste management in Kisumu, Kenya.
• Scenarios involve a waste-to-biogas initiative and a ban on open burning in landfill.
• Combined scenario generates 1.1m tonnes cumulative GHG savings by 2035.
• Largest contribution (42%) is from biogas substituting traditional cooking fuels.
• Combined scenario may save 1,150 cumulative life years by 2035, plus ~220 more p.a.

## Graphical abstract

**Figure:**
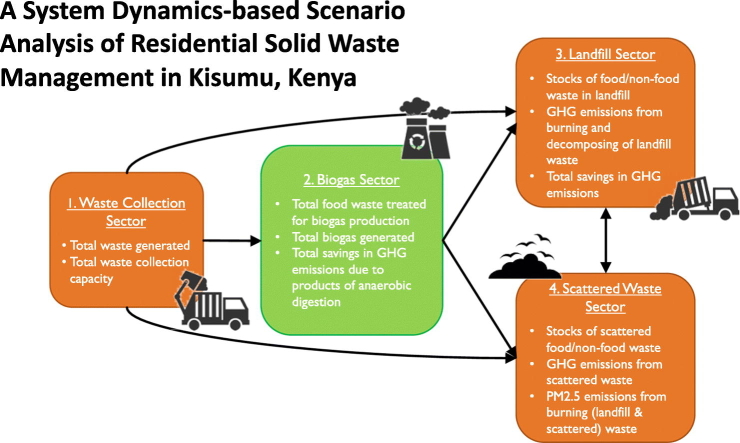

## 1 Introduction

Municipal solid waste management (MSWM) in sub-Saharan Africa (SSA) remains a critical challenge despite the development of several continent-wide and regional policies and strategies to address this (see for example African Union Commission, 2015, EAC, 2016, WHO, 2018). With projected population growth, rapid urbanization and economic growth, production of solid waste is expected to increase, and this, coupled with weak implementation of existing legislation and budgetary constraints for waste services, may worsen the situation (UNEP, 2018). In most cities in the region, open dumpsites (both controlled and uncontrolled) are the final resting place of the collected municipal solid waste (MSW), posing environmental and health challenges for city dwellers (UNEP, 2018). Emissions of climate changing greenhouse gases (GHG) occur at various stages across the SWM service chain. Across many African cities, waste collection and transportation fleets are old, leading to higher emissions of GHGs (Friedrich and Trois, 2011). In addition, with the prevalence of open dumpsites without gas harvesting systems, the decomposition of organic waste leads to the release of methane (Friedrich and Trois, 2011). This gas can, however, be harnessed as an alternative and clean source of energy for the more than half of households in SSA who rely on biomass and kerosene for cooking (Lambe et al., 2015; Morrissey, 2017). Biomass fuels as well as kerosene have been associated with high emissions of household air pollutants with implications for the health of users and their families (WHO, 2021). With the 2030 deadline of the SDGs—including SDG 7 on access to clean affordable energy—less than a decade away, governments in Africa and elsewhere where biomass is a dominant fuel must find alternative clean fuels for households. Via exploring the potential of a proposed waste-to-biogas initiative in providing energy for cooking, in reducing GHG emissions, and in improving air quality and associated health outcomes, this paper provides a unique opportunity in the search for pathways towards affordable and clean energy in Kisumu County, Kenya.

**Fig. 1:**
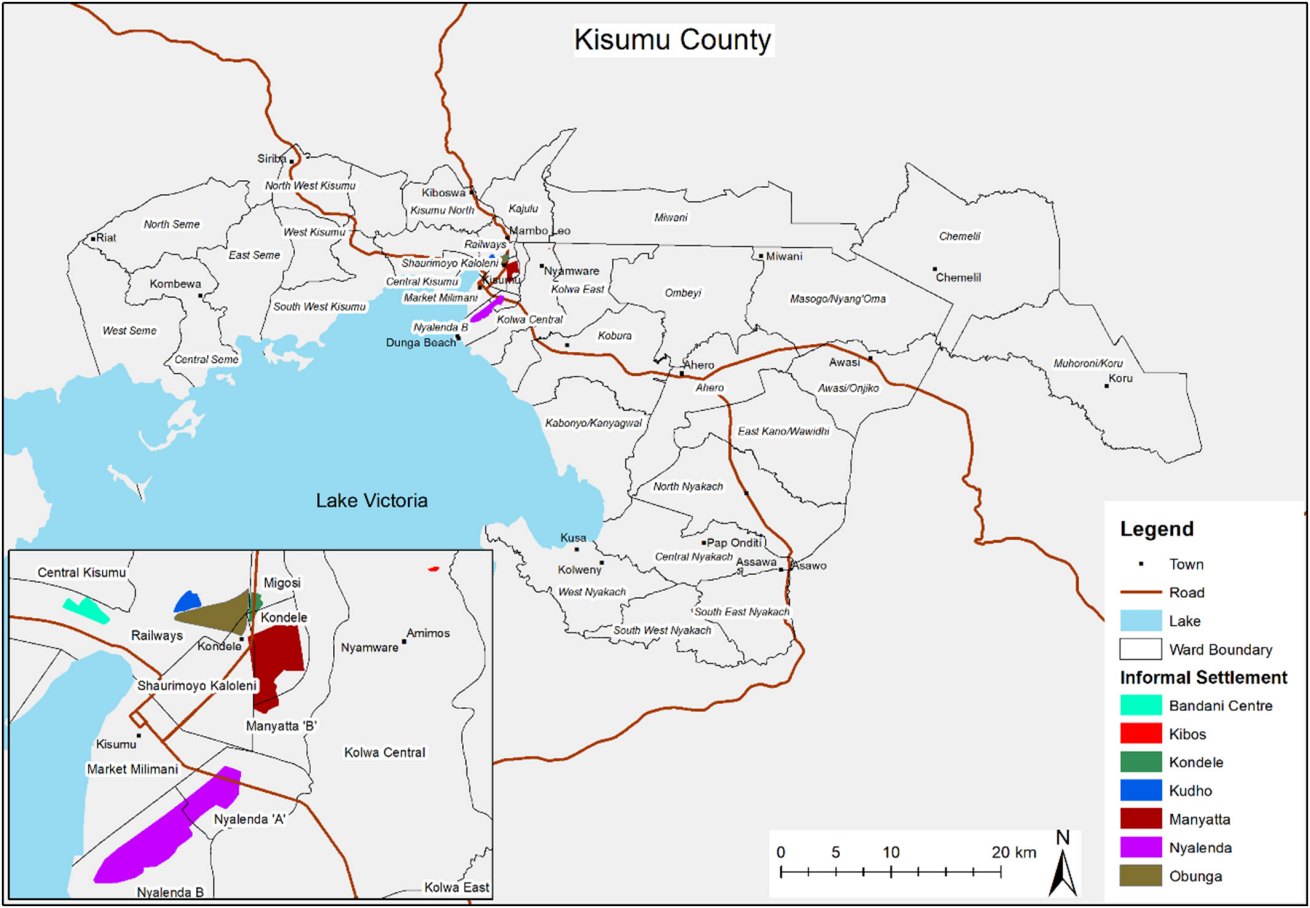
Map of Kisumu County.

Source: African Population and Health Research Centre

Kisumu County, which has Kisumu City as its capital, is strategically positioned in the west of Kenya on the shores of Lake Victoria (Fig. 1), the second largest freshwater Lake in the world. Kisumu, the third largest city in Kenya, is a key commercial and transport hub for the Western region of Kenya and the East African region. In spite of that, over half of Kisumu City's population are categorised as poor (Olang et al., 2018), and the County scores 0.49 on the Human Development Index (HDI), below the national average at 0.56 (County Government of Kisumu, 2019). The 2019 population census indicates that the county has a population of about 1,156,000 people (KNBS, 2019). Population has been growing at a rapid rate of about 2.3% per year and is expected to continue to grow at over 2% per year until 2030 (United Nations, 2019).

Rapid urbanization and changing consumption patterns, together with poor environmental management, have turned MSW into an alarming crisis for Kenya, manifest in the commonly overflowing dumpsites in the cities which are cause for environmental and health hazards (Awuor et al., 2019). As with many urban areas in the Global South, Kisumu is struggling with an overflowing dumpsite as well as consequent environmental and health risks associated with improper disposal of MSW (Sibanda et al., 2017). Kisumu County generates about 500 t of solid waste per day^1^ (Oyake-Ombis, 2017) out of which, based on estimates we obtained from local actors in the system, only about 40% is collected for disposal at the city's open landfill (see Appendix A, Section i). Other estimates indicate even lower shares, starting from as low as 10–20% (Aguko et al., 2018; Awuor et al., 2019).

**Fig. 2:**
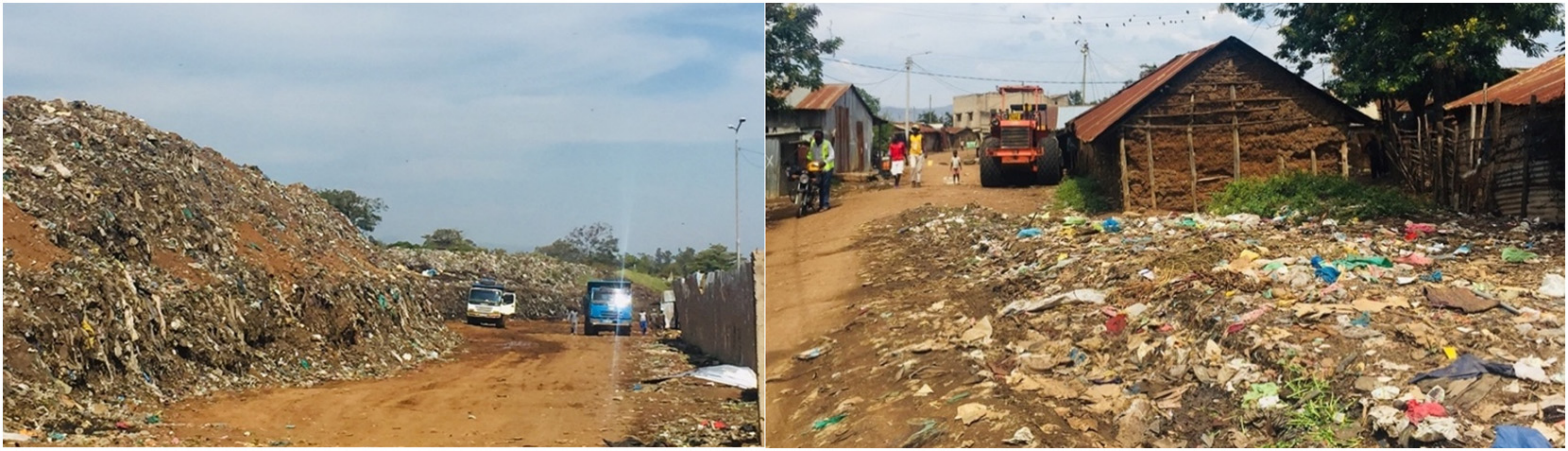
Kachok dumpsite (left) and roadside scattered waste in Nyalenda slum (right).

Kachok dumpsite (Fig. 2 left photo), located within the city's central business district and only 2 km from the centre, has accumulated the city's waste since 1975 (Awuor et al., 2019). The dumpsite is about 2.7 ha and is characterised by open burning of waste—aimed at reducing the volume of waste and preserving disposal space at the site (Awuor et al., 2019)—as well as noise, odour from decaying organic matter, dust, and smoke. There are also concerns around insecurity, public health, and environmental degradation due to the pollution of Lake Victoria through leachate run-off which typically contains heavy metals, organic pollutants and microbial pathogens (County Government of Kisumu, 2017; Sibanda et al., 2017; Tyagi et al., 2018). Uncontrolled open dumping and open burning of waste contribute to the emission of climate altering GHGs such as methane (CH_4_), as well as carbon dioxide (CO_2_) and black carbon (BC). In addition, the open burning of waste also generates toxic air pollutants such as fine particulate matter into the air which cause respiratory, cardiovascular and other kinds of diseases when inhaled (Sibanda et al., 2017). Aguko et al. (2018) report higher concentrations of such air pollutants over and around Kachok dumpsite (Aguko et al., 2018). Efforts towards relocating the overflowing dumpsite to a larger site farther away from the city centre have so far not been successful. In a comprehensive review of the state and history of Kachok dumpsite, Awuor et al. (2019, p. 4) make the following observation: “[i]n its location and current state, [Kachok dumpsite] is an environmental and health hazard defeating the purpose for waste disposal sites; which is to protect human and wildlife populations from health hazards and the environment from degradation.”

Photo credit: Aarathi Prasad

The lion's share of the city's waste remains uncollected and accumulates in skips (or where skips used to be), is openly burnt, illegally dumped on vacant land, alongside roads (resulting in numerous unsightly garbage heaps scattered around the city, see Fig. 2 right photo) or in drainage systems (resulting in frequent flooding of neighbourhoods with waste and sewage water) (Gutberlet et al., 2017; Sibanda et al., 2017). The County has developed and revised an *Integrated Solid Waste Management Plan (KISWAMP)* (County Government of Kisumu, 2017), but this has so far failed to result in a transformation of the state of MSWM in Kisumu (Awuor et al., 2019).

In line with Kenya's strategic target of reducing GHG emissions by 30% by 2030, as pledged at COP-21 in Paris, 2015 with a strong focus on increasing access to renewable energy (Dalla Longa and van der Zwaan, 2017), Kisumu County's *KISWAMP* (County Government of Kisumu, 2017) discusses the potential in waste-to-energy (WtE) technologies. Currently, a wide range of such technologies exist. These are broadly categorised as thermal (e.g., incineration, pyrolysis, gasification) and biological (e.g., aerobic composting or anaerobic digestion/biogasification) (Moya et al., 2017). We assert that incineration, which is the most widely used method (Fernández-González et al., 2017), is not suitable for the context of Kisumu primarily because the composition of waste in Kisumu, which consists of over 60% biowaste (Sibanda et al., 2017), as is common in low-income settings (Hoornweg and Bhada-Tata, 2012; Kumar and Samadder, 2017), negatively affects its calorific value and impairs the practicability and energy efficiency of thermal treatment options which are more suitable for low-moisture waste (Dlamini et al., 2019; Franca and Bassin, 2020). Secondly, incineration requires very large waste input to be viable and is more suited to areas of higher population (Fernández-González et al., 2017). It is also very capital-intensive, estimated by Kisumu County to require around $20 M of initial capital investment (County Government of Kisumu, 2017, p. 54) and also involves high operating costs (Tyagi et al., 2018). Lastly, there are important environmental and health concerns around incineration, as it may emit various particulate and gaseous pollutants (Kumar and Samadder, 2017; Tyagi et al., 2018; Istrate et al., 2020). Other advanced thermal treatment processes such as pyrolysis and gasification are deemed to be “technically challenging, relatively unproven at commercial scale, and […] the generated energy may be needed to power the process (Tyagi et al., 2018, p. 381).”

The same mostly organic composition of Kisumu's waste, however, makes it highly suited for biological treatment (Gebreegziabher et al., 2014). Anaerobic digestion (AD) is the biological decomposition of organic waste in an oxygen deficient environment (Dlamini et al., 2019), which turns the ‘biowaste’ into two valuable products: (a) energy-rich renewable biogas, a methane-rich gas produced by biological means, and (b) nutrient-rich digestate which can be used directly or after composting in agriculture (Tyagi et al., 2018). As it entails relatively lower capital investment compared to thermal treatment options, AD is also considered the most feasible MSWM solution in low-income countries (Kumar and Samadder, 2017), with various studies asserting that it holds significant promise in SSA for helping to mitigate the problems of urban waste management, energy insecurity and climate change (Abila, 2014; Gebreegziabher et al., 2014; Dlamini et al., 2019; Franca and Bassin, 2020). Biogas technology helps mitigate climate change by reducing GHG emissions, both via substituting fossil fuels for cooking, heating, lighting, or electricity generation, and via avoiding emissions associated with mineral fertiliser production (Gebreegziabher et al., 2014). There is generally a consensus on the favourable environmental consequences of the diversion of organic waste from aerobic, GHG emitting composting to anaerobic digestion (Istrate et al., 2020).

In Kenya, in the city of Naivasha, 76 km from Nairobi, a 2.4 MW commercial biogas plant, with a cost of $6.5 million and an annual treatment capacity of 50,000 t of organic waste, inaugurated in 2017 and is reportedly the largest grid-connected biogas power plant in Africa, meeting the power needs of 6000 rural homes (Roopnarain and Adeleke, 2017; Kemausuor et al., 2018). In this paper, however, rather than proposing to use biogas from waste to generate electricity, we explore the option of making the biogas directly accessible to households for use in cooking. Currently, close to 80% of households in Kisumu use traditional biomass fuels (mainly wood and charcoal) for cooking (KNBS, 2019, p. 336). Indoor air pollution caused by traditional cooking is today's most important environmental health risk and second-largest risk factor in all categories in Eastern SSA (Lim et al., 2012). Women and children are disproportionately at risk of health issues caused by indoor air pollutants. Furthermore, the use of wood and charcoal for cooking is a major driver of deforestation and GHG emissions (Carvalho et al., 2019). Evidence shows that using alternative cook stoves significantly reduces indoor air pollution, and numerous studies demonstrate the link between reductions in household air pollution and improved respiratory health (Anderman et al., 2015). Tumwesige et al. (2017) monitored real-time PM2.5 and CO concentrations in 35 households in Cameroon and Uganda where biogas and firewood (or charcoal) were used and found that fully switching to biogas for cooking reduces both CO and PM2.5 concentrations to below WHO recommended limits. Although no direct evidence on the health benefits of households switching to biogas is available, comparable studies of households switching to LPG suggest that such a shift could bring respiratory and cardiovascular health benefits of the order of 20–25% reduction in risk of a wide range of diseases (Semple et al., 2014). Within the context of Kisumu, Carvalho et al. (2019) compare the results of four biomass cookstove strategies on reducing energy consumption and air pollutant emissions in Kisumu County and find that, at least in the medium-term, the highest energy savings, as well as reductions in GHG, PM_2.5_ and BC emissions and the accompanying burden-of-diseases, in comparison to business-as-usual, are achieved via a transition to biogas cookstoves (Carvalho et al., 2019). Currently, 18.7% of households in Kisumu use LPG for cooking, versus less than 1% using electricity (KNBS, 2019), testament to the higher degree of readiness for the uptake of gas-burning cookstoves versus electric ones. Furthermore, electricity generated from biogas plants would have to compete with low-priced (often subsidised) electricity from other sources, while electricity generation from biogas is relatively expensive, even with free substrates, especially in countries where the technology is imported (Kemausuor et al., 2018). The above considerations justify the choice to use the biogas directly for cooking rather than for electricity generation.

In Kenya, there are already numerous small-scale biogas installations in operation (Kemausuor et al., 2018), including in Kisumu (Sibanda et al., 2017). Within the Africa Biogas Partnership Program, which aimed to promote adoption of biodigesters by rural households in SSA, over 27,000 households in Kenya, Tanzania and Uganda installed a biodigester between 2009 and 2017, half of which in Kenya (Clemens et al., 2018). In fully replacing traditional cooking fuels by clean biogas, Kenya showed the highest success, with half of the adopters exclusively using biogas, while the other two countries reported higher rates of fuel stacking, i.e., using a mix of fuels rather than a complete transition to biogas. Clemens et al. (2018) suggest that the Africa Biogas Partnership Program has succeeded in creating a nascent biodigester market in East Africa, but challenges such as high upfront cost, limited access to credit, and lack of maintenance still remain. Similarly, Sibanda et al. (2017) maintain that technical knowhow and financial investment in this area is limited and further capacity building is needed (Sibanda et al., 2017).

In summary, it appears that anaerobic digestion of biowaste to produce biogas for use in household cooking holds great potential in reducing waste to landfill and associated externalities (e.g., pollutant and GHG emissions, groundwater contamination), while simultaneously improving indoor air quality and related health outcomes. Within this context, the purpose of this study is therefore to explore the idea of a transition towards anaerobic digestion of Kisumu's organic fraction of MSW and the use of the produced biogas in household cooking on the levels of waste accumulating in landfill or waste scattered elsewhere, on waste related GHG emissions, on air pollutant concentrations, and on related health impacts. The novelty and importance of this paper lies in the quantification of a potential waste-to-biogas scenario and its environmental and health impact in Kisumu for the first time.

Existing studies on the impacts of WtE technologies in other contexts—e.g., Ayodele et al. (2017) in Nigeria, Chaya and Gheewala (2007) in Thailand, Evangelisti et al. (2014) in the UK, and Rigamonti et al. (2010) and Cremiato et al. (2018) in Italy—tend to take a static Life Cycle Assessment (LCA) approach. Considering that the waste system involves distinctly dynamic processes, such as the accumulation, depletion and degradation of stocks of waste, static methods do not appear up to the task of informing policymaking in this area, where investments are often large-scale with long timeframes in mind. Thus, for various reasons, the primary method used in this study is system dynamics (SD). Firstly, a key advantage of SD over common spreadsheet waste management models such as LCA is the dynamic nature of SD models, versus the static optimization in spreadsheet-based methods (Adamides et al., 2009; Inghels and Dullaert, 2011). Secondly, it not only allows to simulate material flows but also captures the decision-making structures managing these flows. Thirdly, SD is a white-box modelling approach, with fully transparent model boundary and assumptions. Fourth, it allows for a visual representation of the underlying system, which enhances the model's communicability. As reviewed later in Section 2.1, SD has been widely applied to problems of MSW around the world.

The rest of the paper is structured as follows. In the next section, the methodology used in this study is described. Subsequently, in Section 3, the results from our scenario analyses are visualised, compared and contrasted. The paper concludes in Section 4 with a brief discussion of the results, implementation challenges and study limitations. This manuscript is accompanied by three Appendices including a full documentation of the model, list of model parameters, and detailed specification of the scenarios. The paper is accompanied by an online supplement containing a folder with the model and all scenario runs.

## 2 Methods

The aims and scope of this study were determined based on a series of eight focus group discussions in Kisumu during July 2019 with representatives from Kisumu County's Department of Environment, the local industry, non-government groups, community-based organizations, academia and resident associations. These discussions, which were audio-recorded and later transcribed, provided context information of the current waste management situation and diverse stakeholder perspectives about it (Salvia et al., 2021). Our scenario definitions were also informed by these discussions.

Multiple methods are combined for the purpose of this study. First, the central method applied is SD (Sterman, 2000), which is introduced in the following Sub-section 2.1. In Sub-section 2.2, a description of the SD model follows. As seen in Appendix B, where all parameter assumptions used in the SD model and their sources are listed, the primary source for parametrising the model has been existing academic papers, national and international databases and industry publications. Data for certain parameters specific to the state of SWM in Kisumu, such as the city's current waste collection capacity or estimates of the current stock of waste in the city's landfill, were obtained in correspondence with the Kachok dumpsite manager and Kisumu county officials.

Second, emission factors used to calculate GHG emissions were obtained according to the IPCC guidelines (IPCC, 2006), as described in Section 2.3. Third, the method for estimating ambient and household PM2.5 concentrations is described in Section 2.4. Fourth, these estimates are fed into a life table health impact assessment model (as described in Section 2.5). This Methods section concludes with a description of our scenarios.

In their review of the main existing approaches to GHG accounting in waste management, including national accounting, corporate level accounting, life cycle assessment, and carbon trading methodologies, Gentil et al. (2009) emphasise the importance of transparency in GHG accounting concerning aspects such as waste type and composition, time period considered, GHGs included, choice of system boundaries, etc. Following this guideline, full transparency is followed in describing the method and the model in the following sub-sections, and in more detail in the Appendices. This being an initial, high-level, aggregate model, it has several limitations, as discussed later in Section 4.3.

### 2.1 System dynamics and its past applications to SWM

System dynamics is a method based on computer simulation where a model of the cause-and-effect relationships of a real-world complex system is built, parametrised and validated using real-world information. The sources of such information can be varied and can include not only those available in numerical datasets and scientific literature, but also those gleaned from the mental models of experts (Forrester, 1987).

Thanks to its strengths in bringing together knowledge from a variety of fields in an integrated framework and in tackling dynamically complex problems, SD has been widely applied to the problem of MSWM in the past. In terms of quality, papers applying SD to SWM are very mixed. The history of such applications goes back around three decades, starting with Mashayekhi (1993) who uses an SD model capturing major interactions between different socioeconomic and environmental factors to study the problem of solid waste disposal in New York. Later, and within the context of a lower-income country, Sudhir et al. (1997) propose an SD model for the study of the potential consequences of various structural and policy alternatives for a sustainable urban SWM system for a typical metropolitan city in India, and conclude by recommending the allocation of waste management funds in proportion to the requirements of collection, disposal and processing, as opposed to prioritising short-term interests such as only collection of waste. Still within the context of India, Talyan et al. (2007) use an SD approach to quantify CH_4_ emissions from MSW disposal under various scenarios in Delhi. Their model shows that an improved waste management system, involving the introduction of composting, biogasification, and refuse-derived fuel, would significantly reduce CH_4_ emissions over time despite an increase in waste generation. Sufian and Bala (2007) build an SD model for SWM in the city of Dhaka, Bangladesh, the results of which show that in order to improve environmental outcomes, it is not sufficient to increase budget for waste collection capacity, but this needs to be accompanied by increasing the budget for treatment, mirroring the finding of Sudhir et al. (1997). This mindset informs the current study as well.

Within the context of Kisumu, Gutberlet et al. (2017) apply a combination of action net theory and systems thinking to build a map of the waste management system in Kisumu with all its actors, actions, processes and interconnections. Their main conclusion is that “new waste initiatives should build on existing waste management practices already being performed within informal settlements by waste scavengers, waste pickers, waste entrepreneurs, and community-based organizations (Gutberlet et al., 2017, p. 106).”

### 2.2 Model description

**Fig. 3:**
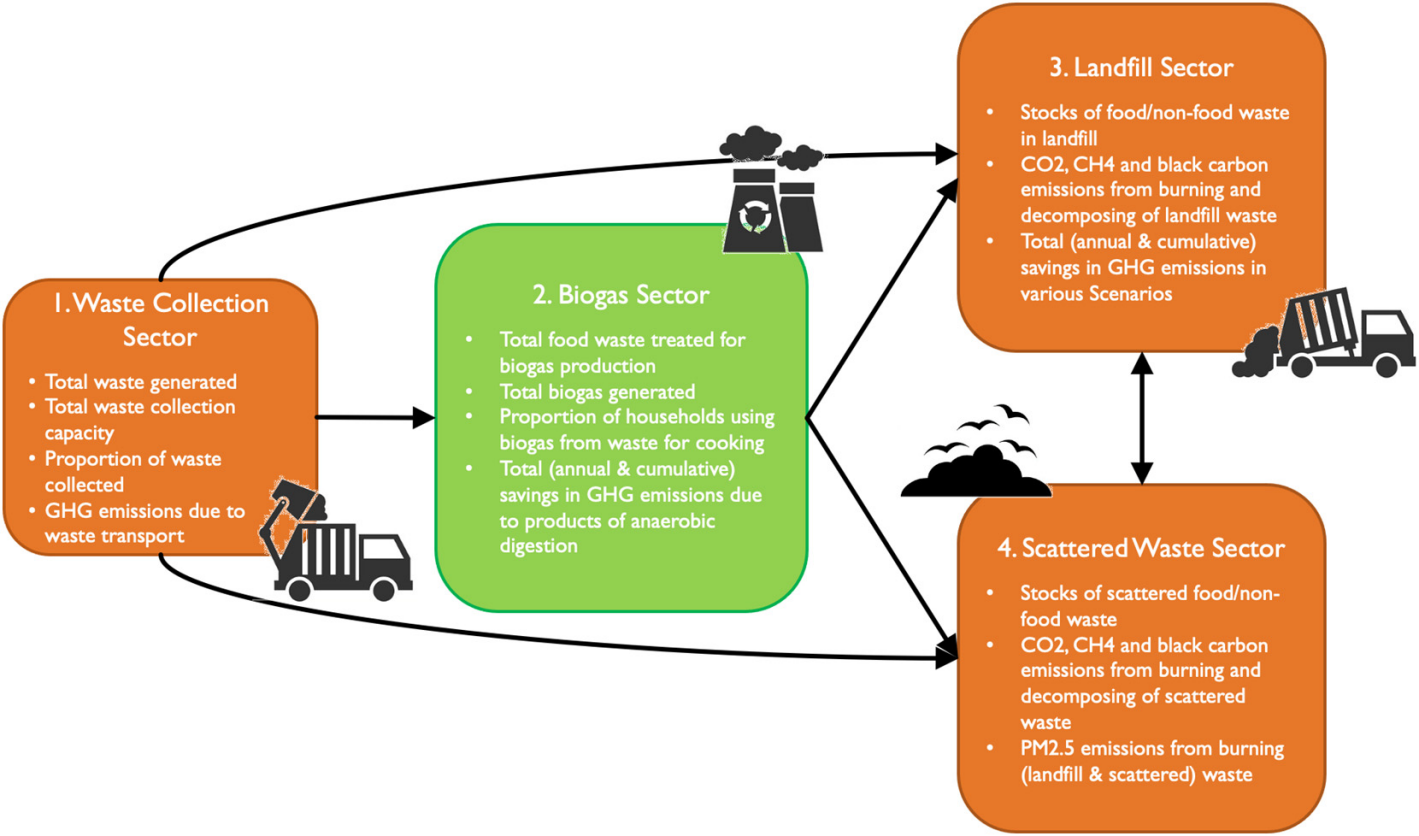
Overview of model sectors and interlinkages.

The full model documentation is provided in Appendix A – Full Model Documentation. In this section, a high-level schematic overview of the model is shown in Fig. 3. The model consists of four inter-connected sectors: (1) Waste Collection, (2) Biogas, (3) Landfill, and (4) Scattered Waste. Variables calculated in one sector are often used as inputs in another sector. In the first sector, which captures waste collection, indicators such as *total waste generated*, *total waste collection capacity*, *proportion of waste collected* and *greenhouse gas emissions due to waste transport* are calculated. In particular, *total food waste collection capacity* becomes a key input to the Biogas Sector, as a constraint on biogas production capacity along with the cumulative capacity of the biogas facilities, together determining *total biogas generated*. Subsequently, the savings in GHG emissions resulting from a switch to clean biogas for cooking are calculated and accumulate in the stock of *cumulative savings in GHG emissions due to products of anaerobic digestion*.

A by-product of the biogas plants is digestate, which can be used as fertiliser, either directly or upon further processing into compost. This organic fertiliser reduces the need for inorganic fertiliser use in the region, potentially countering another source of GHG emissions. However, there is substantial uncertainty around the extent of such savings (Møller et al., 2009). Cecchi et al. (2011) estimate these savings in the range of 30–40 kg-CO_2_t^−1^ while cautioning that fugitive CH_4_ and N_2_O emissions when digestate is applied on land, ranging from 0 to 50 and from 30 to 60 kg-CO_2_t^−1^ respectively, can cancel out any savings (Cecchi et al., 2011). The aggregate result will depend on the exact operating conditions and is likely to be small (Møller et al., 2009). Therefore, any digestate-related GHG saving or load is disregarded in this model. Similarly, assuming that any fugitive CH_4_ emissions from the biogas plant are flared, such emissions are not accounted for.

Next, the waste that remains and that is not used for biogas production is transported to landfill, as captured in the Landfill Sector, given our mixed waste collection constraints (coming from the Waste Collection Sector). The accumulation of food and non-food waste in landfill, together with any reductions in the waste mass via open burning, natural decomposition and informal waste-picking are captured in the Landfill Sector. Furthermore, emissions of different types of GHGs as a result of burning and decomposition, including carbon dioxide (CO_2_), methane (CH_4_) and black carbon (BC), are also calculated, along with the annual and cumulative savings in GHG emissions (both from landfill waste and from scattered waste, as imported from Scattered Waste Sector). Various emission factors for food and non-food waste required for these calculations are derived based on best available evidence, as described in Section 2.3. A key feature of the model is that the food and non-food contents of the waste that remains after biogas production and is disposed of are dynamically calculated. This leads to outcomes which are not immediately evident without using simulation, as we will see in the results (Section 3).

Similarly, the Scattered Waste Sector captures the accumulation, depletion and emission processes for food and non-food waste which is not collected due to the constraints of our waste collection fleet capacity and is structured in the same way as the Landfill Sector. Besides GHG emissions, particulate matter (PM_2.5_) emissions from both landfill and scattered waste are also calculated in this sector, which are then used for estimating the potential effects of our scenarios on population health, according to the method described in Section 2.5.

With regards to the boundaries of the model, based on Gentil et al.'s (2009) proposed upstream-operating-downstream framework for GHG accounting in waste management, in the ‘indirect upstream’ category, in the model we have accounted for emissions from waste transport; in the ‘direct operating’ category, we have accounted for landfill and scattered waste emissions (CH_4_ from decomposition and CO_2_ and BC from burning), and in the ‘indirect downstream’ category, we have accounted for savings resulting from the biogas substituting biomass in household cooking. These boundaries for the model can be considered in compliance with Møller, Boldrin and Christensen's (2009, p. 823) conclusion that “irrespective of the employed technology, as long as the produced biogas is utilized for energy substitution, the indirect downstream emissions are the most important factor. Direct emissions at the AD facility and indirect upstream emissions play less important roles.”

### 2.3 Development of emission factors

We use emission factors from the GAINS model (Amann et al., 2011, Amann et al., 2020) in our analysis. Methane emission factors and carbon flows follow Gómez-Sanabria et al. (2018) and are developed in line with the method presented in the IPCC Guidelines (IPCC, 2006, vol. 5 ch. 3). Both are representative of the particular waste composition in Kisumu from County Government of Kisumu (2017). Following the local waste management conditions, the estimated emission factor for landfill food waste is 20.27 t CH_4_/kt dumped food waste. For the non-food waste fraction, the emission factor is estimated at 17.70 t CH_4_/kt dumped non-food waste. For scattered waste, emission factors for food waste and non-food waste are estimated to be 10.13 t CH_4_ /kt and 8.85 t CH_4_ /kt, respectively.

Furthermore, the method suggested in the IPCC Guidelines (IPCC, 2006, vol. 5 ch. 5) is applied to estimate CO_2_ emissions from open waste burning. Emission factors are calculated for each fraction of waste based on the fossil carbon content. CO_2_ emissions from biogenic origin are not included in the estimates as advised in the IPCC Guidelines (IPCC, 2006, vol. 5 ch. 5). This means that CO_2_ emissions from open burning of food and wood waste are set to zero. The implied CO_2_ emission factor for open burning of the non-food waste fraction in Kisumu is 464.89 t CO_2_/kt of waste burnt.

Emission factors for black carbon (BC) and PM_2.5_ are adopted from Akagi et al. (2011) and Christian et al. (2010) and are in line with the emission factors used by Klimont et al. (2017) and Wiedinmyer et al. (2014). The emission factors are 8.74 t/kt waste burnt for PM_2.5_ and 0.65 t/kt waste burnt for BC. These emission factors are for mixed waste and are not representative of Kisumu's particular waste composition.

Table 1 presents estimated CH_4_ and CO_2_ emission factors for the Kisumu waste composition.

**Table 1:** CH_4_ and CO_2_ emission factors.

Item	Unit	Food waste	Non-food waste
CH_4_ emission factor for scattered waste	tonne CH_4_/kt	10.13	8.85
CH_4_ emission factor for landfill waste	tonne CH_4_/kt	20.27	17.70
CO_2_ emission factor for burnt waste	tonne CO_2_/kt	n/a	464.89

Table 2 shows the background information needed to carry out the estimation of the emission factors.

**Table 2:** Estimation of emission factors.

Kisumu waste composition	Composition in %	kt waste	Dry matter content in % of wet waste	DOC % in dry waste	Fossil carbon content in % of total carbon	Total CC in % of dry waste
Food waste	0.636	49.56	40	38	0	38
Paper	0.122	9.51	90	44	1	46
Plastic	0.102	7.95	100	0	100	75
Glass	0.032	2.49	100	0	0	0
Scrap Metal	0.013	1.01	100	0	0	0
Other	0.095	7.40	90	0	100	3

### 2.4 Estimation of ambient and household PM_2.5_ concentrations

The PM_2.5_ annual emissions obtained based on the above emission factor are converted into ambient PM_2.5_ concentrations using a simplified version of the atmospheric calculations in the GAINS model (Amann et al., 2020) which themselves rely on a linearized representation of full atmospheric chemistry transport model simulations. GAINS contains atmospheric transfer coefficients from all source pollutants for PM_2.5_ in Kenya to a 0.1° receptor grid. As detailed in Appendix A (Section iv), we developed an integrated atmospheric transport coefficient from near-ground emissions of PM_2.5_ in Kisumu to ambient PM_2.5_ concentrations in Kisumu, which is then applied to the respective emissions from residential combustion and MSW burning to estimate their impacts.

For household PM_2.5_ concentrations, we used an approximation method with a high level of uncertainty, described in detail in Appendix A (Section ii), which is based on empirical measurements reported in Muindi et al. (2016, p. 7 Table 3) on mean levels of indoor PM_2.5_ concentrations in households using different cooking fuel types.

### 2.5 Health impact assessment

We estimated the effect of changes in exposure to ambient and household PM_2.5_ on mortality in Kisumu under each scenario using life tables based on the IOMLIFET model (Miller and Hurley, 2003) programmed in R (version 3.5.1, R Foundation for Statistical Computing, MA, USA). The effects of changes in PM_2.5_ were modelled by applying to the life tables the Global Burden of Disease (GBD) Integrated Exposure-Response functions relating long-term PM_2.5_ exposure to mortality risk from five causes – ischaemic heart disease (IHD), chronic obstructive pulmonary disease, stroke, lung cancer and lower respiratory infections (LRI) (Apte et al., 2015). The functions for IHD and stroke varied by age.

The life tables were set up using age- and gender-specific population and cause-specific mortality data for Kenya from the GBD's GHDx tool for the closest available year of data to the study period (2017). The national-level population data was downscaled to represent the population of Kisumu. Single-year-of-age mortality rates were calculated from 5-year rates via one-way spline interpolation using the MS Excel add-in, SRS splines (version 2.5, SRS1 Software LLC, MA, USA).

We combined ambient and household PM_2.5_ as a time-weighted average, assuming that men and women in Kisumu spend 50% and 80% of their time indoors at home, respectively. To account for delays in changes in mortality risk following air pollution exposure reductions, we incorporated cessation lags for each outcome. These were exponential functions parameterised using evidence from studies of smoking cessation (Lin et al., 2008) and assumptions about disease progression over time. For IHD and lung cancer, we assumed the full effect would be reached after 15–20 years, with shorter lags for COPD, stroke and LRI.

The outputs from the life tables are life years lived by the population over the study period. Solid waste may give rise to other forms of adverse health impact but in the analysis presented in this paper, we concentrate only on those arising from contamination of the outdoor air by fine particles (PM_2.5_) arising from burning of solid waste.

### 2.6 Description of scenarios

In this study, we simulate four different scenarios as summarised in Table 3. The scenarios were developed in close connection to planned developments of Kisumu City regarding waste management strategies (County Government of Kisumu, 2017) and designed to account for local structural factors as well as international guidelines.

**Table 3:** Summary of scenarios.

No.	Scenario name	Waste collection fleet	Biogas production capacity	Ban on landfill waste burning
(1)	*Baseline*	Slow gradual increase in mixed waste trucks (one additional truck every two years)^a^	–	No.
(1b)	*Ban on Burning*	Same as above.	–	Yes. Over 8 years.
(2)	*Biogas*	New organic waste handcarts from 83 units (49.3 t per day = 18 k tonnes/year) in 2022 gradually up to 411 units (246.6 t per day = 90 k tonne/year) in 2028.	From 18,000 t/year (six facilities) in 2022 up to 90,000 t/year (30 facilities) in 2028.	No.
(2b)	*Biogas + Ban on Burning*	Same as above.	Same as above.	Yes. Over 8 years.

a All other scenarios include this baseline assumption.

In our *(1) Baseline* (business-as-usual) scenario, we assume only a gradual increase in the mixed waste collection transport fleet, in line with recent trends. Waste volume at the dumpsite is mainly managed through open burning (as the existing mechanical compactor is insufficient and usually non-operational due to inadequate maintenance). At the same time, since most of the waste is composed of moist organic matter, combustion occurs only on the surface and does not significantly reduce waste volume (Awuor et al., 2019). This open burning is a major contributor to emissions of GHGs and atmospheric pollutants (Forbid et al., 2011). In scenario *(1b) Ban on Burning*, we assume the enforcement of a gradual regulatory ban on the open burning of waste in landfill.^2^ The ban on open burning is one of the *Global Waste Management Goals* set out by the United Nation's Environment Programme (UNEP) in the Global Waste Management Outlook (Wilson et al., 2015). This being a major change in SWM practices in Kisumu, in consultation with local county officials we assume that the *Ban* takes place over an extended period of eight years, bringing the fraction of waste annually burnt in landfill from the current 23% per year (Onyango and Kibwage, 2008) gradually down to zero. This *Ban* is assumed to be enforced only in dumpsite at this stage.

In the *(2) Biogas* scenario, we assume a phased commissioning of 30 decentralised biogas facilities in different locations in Kisumu County over a period of eight years (about four new facilities each year). Each facility is envisaged as a medium-sized plant with a treatment capacity of 3000 t of biowaste per year (roughly 8 t per day), taking the total cumulative capacity up to 90,000 t annually, roughly two thirds of Kisumu's food waste, by 2028. The plants are proposed to be commissioned gradually so that the required funding becomes less prohibitive and can be provided in installations and so that learning from commissioning and operation of plants can be transferred from each phase to the next. These are envisaged to be medium-sized facilities with trained staff, with the intention of avoiding dis-adoption of the technology reported to often take place in household-level initiatives as a result of technical problems and untrained users (Clemens et al., 2018). Such a program is compatible with existing mindset in the County Government. In one of our focus group discussions with representatives from the local government one County official said the following:

“even if we cannot get one big plant to produce that amount of biogas or energy, can we use this devolved system so that every sub-county has a place where we can drive and dispose of the organic waste.”

It is also in line with Gebreegziabher et al.'s (2014) recommendation of communal or institutional level installations as the ideal scale for overcoming the infrastructure challenges of biogas. Biogas initiatives of a similar scale have already been successfully implemented elsewhere in the developing world, e.g., the *Valorgas* project in India (VALORGAS, n.d.).

In terms of substrate provision, these plants would need to be supplied with source-separated organic fraction of MSW. The decentralised approach has the advantage of minimising the distance travelled for transporting the waste to treatment facilities (Gebreegziabher et al., 2014). We assume that a separate collection system for food waste is gradually built up to match the plants' expanding waste treatment capacity. The collection and transportation of the food waste shall be done by special-purpose handcarts, capable of accessing narrow alleyways in the informal settlements and operated by waste collectors formally employed by the City—perhaps recruited from among current informal actors in the sector, in line with Gutberlet et al.'s (2017) context-specific recommendation of building improved SWM practices on existing ones.

As outlined and justified earlier in the Introduction, we assume that the produced biogas will then be bottled and distributed to households at filling stations for use in cooking instead of currently prevalent biomass and kerosene (KNBS, 2019, p. 336). A distributed set of facilities makes the filling stations more easily accessible for households while providing jobs to the local community. Based on the assumption of a 3000 t per year treatment capacity, a yield of 100 m^3^ per tonne of food waste (Veeken (2005) cited in Müller (2007, p. 26, Table 3)), and an average household need of 262.5 m^3^ biogas per year for cooking (see Appendix B for sources and calculation), each facility is expected to provide cooking fuel for around 1150 households. A recent working paper by Twinomunuji et al. (2020) suggests that, in the SSA region, biogas-based cooking fuels would compete favourably in price with other commercial fuels, including LPG. While highlighting the promise in such initiatives, they furthermore identify several barriers towards widespread interest in bottled biogas in Africa, which will be discussed later in Section 4.2.

Finally, in scenario *(2b) Biogas + Ban on Burning*, we combine the abovementioned assumptions of scenarios *1b* and *2*. In all scenarios we assume a growth in the number of households in line with the growth rates in the United Nation's Probabilistic Population Projections (median variant) (United Nations, 2019).

## 3 Results

In this section, we use simulation to gain insight into the likely future developments in the dynamics of waste accumulation, associated GHG emissions, PM_2.5_ concentration and consequent health outcomes under the described sets of scenario assumptions. We will start by comparing projected trends in waste accumulation under the *Baseline* and *Biogas* scenarios in the first sub-section and continue by comparing GHG emissions under the two scenarios in the following sub-section. Next, we will look at results from the *Ban on Burning* scenario and the *Combined* scenario. The last two sub-sections deal with projections related to changes in PM_2.5_ and the resulting health impacts.

### 3.1 Stocks of waste: *Baseline* and *Biogas* scenarios

**Fig. 4:**
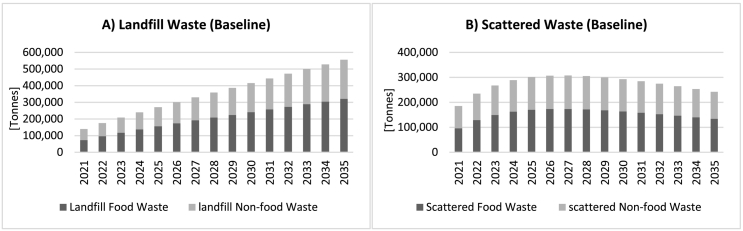
Baseline simulation: developments in A) landfill waste and B) scattered waste.

Fig. 4 shows simulated developments in the stocks of waste under the *Baseline* scenario. As can be seen, landfill waste (both food and non-food components) keeps increasing, reaching over 500,000 t by 2035, as a result of population growth along with a gradual increase in the city's fleet of mixed waste trucks. *Scattered Waste*, on the other hand, starts rising initially, peaks at just over 300,000 t around 2027 and gradually falls thereafter, down to about 242,000 t by 2035. This is because of the assumption of a gradual expansion in the waste collection fleet which eventually overtakes the slow growth in population, with the *proportion of waste inappropriately disposed of* (not shown here) going down from around 57% in the beginning to around 22% over the 15 years of the simulation period.

**Fig. 5:**
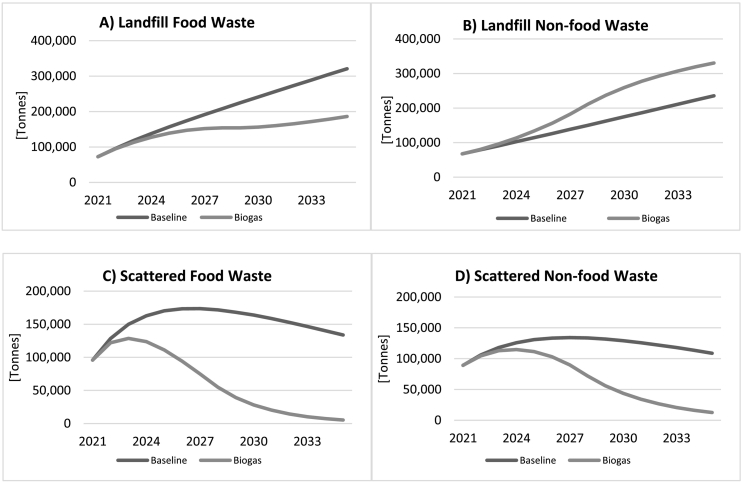
Stocks of waste, Baseline vs. Biogas scenario. A) Landfill food waste; B) Landfill non-food waste; C) Scattered food waste; D) Scattered non-food waste.

Fig. 5 portrays developments in the four stocks of waste under the *Baseline* and *Biogas* scenarios. The top two graphs show projected developments in food and non-food waste in landfill, while the bottom two graphs show projected developments in scattered waste. Regarding landfill waste, both food and non-food components increase in a linear fashion under the *Baseline* scenario. As for the *Biogas* scenario, *landfill food waste* is projected to reach less than 60% of its *Baseline* value by 2035. This is not surprising because as more and more of the food waste (57% by 2035) is used for biogas production, there is less food waste being transported to landfill, to the point that the flow of food waste into the stock comes close to the aggregate outflows due to decomposition and burning, keeping *landfill food waste* relatively stable. Conversely, there is a relatively higher accumulation of non-food waste in landfill, as the waste that is left after biogas production to be transported to landfill becomes more non-organic in nature, with the non-food content ratio (not shown here) going from around 37% initially to 58% by the end of the simulation period in the *Biogas* scenario, while it stays roughly constant in the *Baseline* simulation.

As for *scattered waste*, both stocks start decreasing after a few years in all simulations, with the decline being much greater under the *Biogas* scenario, where *scattered waste* reaches near zero by the end of our simulation period. The improvement in accumulated *scattered waste* under the *Baseline* scenario is a result of an assumed gradual expansion in the city's waste collection fleet where it is assumed that one truck is added to the mixed waste collection fleet every two years. In the *Biogas* scenario, on top of this we have an assumption of a fleet of special-purpose food waste handcarts coming into operation. This increases total waste collection capacity to 100% of the waste by 2028 and leaves zero inflow to the stocks of *scattered waste*. It takes several more years, however, for the already existing *scattered waste* to completely vanish as a result of either natural decay or open burning.

### 3.2 Greenhouse gas emissions: *Baseline* and *Biogas* scenarios

**Fig. 6:**
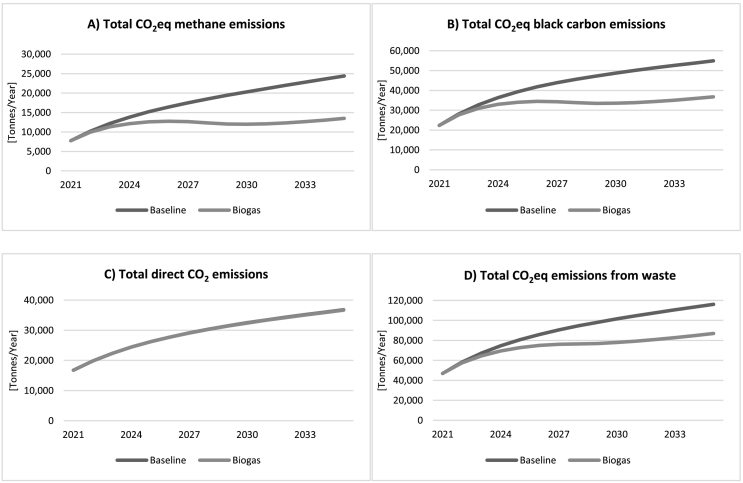
Comparison of emissions *Baseline* vs. *Biogas* scenario.

Projected GHG emissions resulting from scenarios 1 and 1b are shown in Fig. 6. The behaviour of *total CO*_*2*_*eq methane emissions* due to waste decomposition (panel A) can be understood by referring to the two graphs on the left hand-side of Fig. 5. With waste being transported increasingly to landfill, landfill waste tends to dominate in determining the behaviour of total CH_4_ emissions, with the CH_4_ emission curves following the curves of accumulating landfill waste in trend, albeit at a slightly slower rate which is a result of the fall in scattered waste. The *Biogas* scenario is expected to cut such emissions down by 45% by 2035, from around 24,400 to around 13,500 t per year. Similarly, black carbon emissions due to waste burning rise at a decreasing rate in the *Baseline* scenario, while they stay fairly stable under the *Biogas* scenarios, cut by about 33% by 2035 as compared to *Baseline*. Since the BC emission factor assumed for all three types of waste is the same, the change in emissions in our scenarios cannot be the result of a redistribution of waste among the various stocks (*food*/*non-food landfill*/*scattered waste*) but is rather the result of a reduction in the sum total amount of the waste that is disposed of due to the recycling of a part of the total waste for biogas production.

On the bottom left (panel C), we can see that *total direct CO*_*2*_ *emissions* due to waste burning do not change in the *Biogas* scenario compared to *Baseline*, with the two curves fully overlapping. This is because, as mentioned in Section 2.3, these emissions are a product of non-food waste only, and total non-food waste does not change under the *Biogas* scenario, rising slowly with population as it does in *Baseline*.

*Total CO*_*2*_*eq emissions from waste* (panel D), resulting from both burning or decomposition, is the sum of the other three variables. Here we see a decrease in total emissions under the *Biogas* scenario of about 25% per year by 2035, from around 116,000 to around 87,000 t per year.

**Fig. 7:**
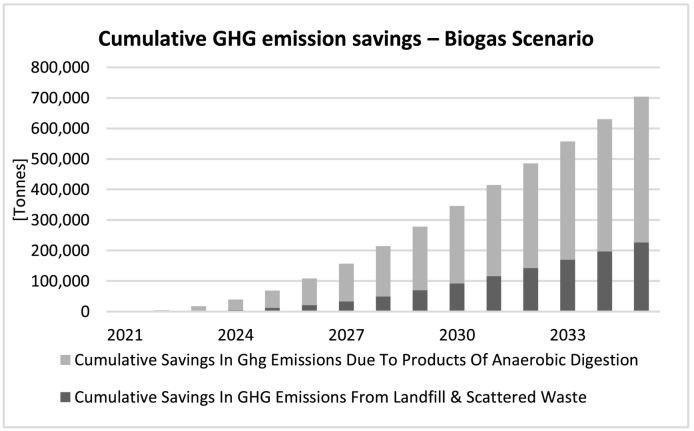
Cumulative saving in GHG emissions under the *Biogas* scenarios.

As a result of this reduction in emissions throughout the 15 years of the simulation as shown in the above figures, as well as many households being able to switch from fossil fuels to renewable biogas for cooking and the resulting digestate from the biogas production process replacing an equivalent amount of inorganic fertiliser, we expect to see a substantial cumulative saving in GHG emissions in the *Biogas* scenario, as shown in Fig. 7. Simulation suggests that by 2035, each year around 9 million m^3^ of biogas can be generated in this way, providing cooking fuel for 8–9% of total households in Kisumu county. Total cumulative savings in emissions reach 700,000 t of CO_2_eq by 2035. Two thirds of these savings come from households switching to biogas, with one third resulting from the reduction of waste in landfill and scattered waste.

### 3.3 *Ban on Burning* scenario

Based on what we saw in Fig. 6, it becomes clear that potentially significant improvements in total emissions are undermined by the lack of any improvements in direct CO_2_ emissions from burning. Therefore, if we are to make more substantial and sustainable improvements in GHG emissions, we need to stop the open burning of landfill waste. Scenarios *1b* and *2b* are envisaged around this assumption. These are the same as Scenarios *1* and *2*, except that in each case a ban on the open burning of landfill waste is gradually enforced, on top of the other assumptions in each scenario. Let us first compare the results of Scenario *(1b) Ban on Burning* with the *(1) Baseline* and *(2) Biogas* scenarios to see how stopping the burning would affect developments in the stocks of waste and the resulting emissions.

**Fig. 8:**
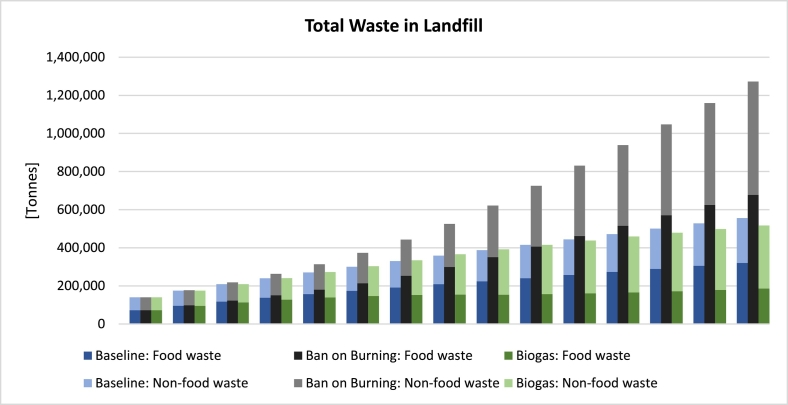
Total waste in landfill: Baseline, *Ban on Burning* and *Biogas* scenarios.

**Fig. 9:**
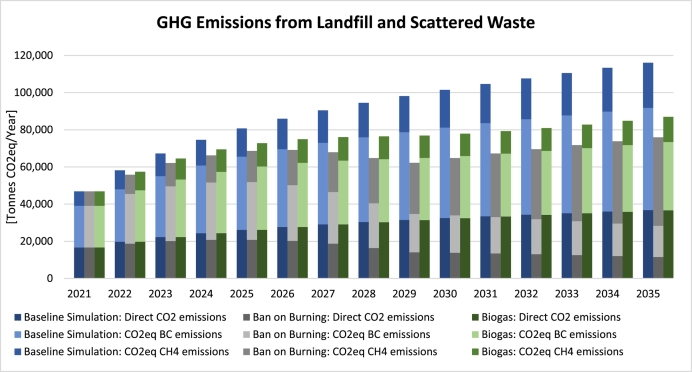
GHG emissions: *Baseline*, *Ban on Burning* and *Biogas* scenarios.

In Fig. 8, the *Baseline* stacks are shown on the left for each year (in blue), the ones for the *Ban on Burning* scenario are in the middle (in grey), and those for the *Biogas* scenario are on the right (in green). Food waste columns are darker in colour, with non-food columns lighter and on top. As can be seen, both types of waste accumulate more rapidly in landfill under the *Ban on Burning* scenario, as open burning constitutes an important way of reducing the mass of waste in landfill and stopping it would lead to waste piling up more rapidly. In total, by 2035, we expect total landfill waste to be 2.3 times higher than the *Baseline* scenario. Mentally simulating the aggregate outcome of this intervention for total emissions is not straightforward because on the one hand landfill waste is growing faster but on the other hand emissions due to burning are reduced to zero in landfill. Simulation can help here by providing a projection for future emissions, as shown in Fig. 9.

In Fig. 9, once again, left-hand side (blue) bars represent the *Baseline* scenario, middle bars (grey) the *Ban on Burning* scenario, and the right-hand ones (green) represent the *Biogas* scenario. The three different types of GHG emissions are distinguished in each column using different colours. As can be seen, in the *Ban on Burning* scenario, CH_4_ emissions due to waste decomposition rise faster, due the quicker accumulation of waste in landfill, as seen earlier in Fig. 8. The other two types of emissions (i.e., CO_2_ and BC emissions due to burning), however, are drastically reduced and, upon the full enforcement of the ban (in 2029), only arise from the burning of scattered waste in places other than the managed landfill. This reduction more than compensates for the increase in CH_4_ emissions, and as a result the aggregate emissions decline notably, standing at about 35% lower than *Baseline* and 13% lower than *Biogas* by 2035. The dynamic behaviour of aggregate emissions under the *Ban on Burning* can be understood in the following way: In the beginning the enforcement is weak and thus aggregate emissions keep rising, albeit behind the *Baseline*. By 2026, aggregate emissions peak as the enforcement of the ban is strengthened. By 2029, a trough is reached as the ban goes into full enforcement, after which aggregate emissions start rising slowly again in line with increases in waste generation, but still more slowly than *Baseline*. Cumulative savings in GHG emissions as a result of this single intervention amount up to 342,000 t by 2035, evidence of how effective the enforcement of such regulation could be in reducing emissions. As for the *Biogas* scenario, with significantly lower dumping of food waste, BC and especially CH_4_ emissions are lower than *Baseline*, with total emissions standing 25% lower by 2035.

### 3.4 Combined scenario

Having seen the significant potential of this intervention for reducing emissions, we will now investigate the expected outcome of combining this with our *Biogas* scenarios, identified as *Scenario 2b* in Table 3. Under the *Biogas + Ban on Burning* scenario, savings as a result of changes in landfill and scattered waste, at 661,300 t CO_2_eq during the 15 years of simulation (~44,000 t per year on average), are drastically higher than the *Biogas* scenario alone at 226,700 t CO_2_eq (~15,000 t per year on average). Cumulative savings in emissions due to the produced biogas is equal in both scenarios, amounting up to around 473,400 t CO_2_eq (~31,500 t per year). Total cumulative savings under the *Biogas + Ban on Burning* scenario amounts up to over 1.1 million tonnes of CO_2_eq over 15 years. Per capita annual GHG emissions in Kenya has been estimated to be 0.41 t CO_2_eq in 2018 (Knoema, n.d.). If we assume current per capita emissions in Kisumu to be approximately at this level, total GHG emissions in Kisumu amounts to around 480,000 t CO2eq per year. Therefore, a cumulative saving of 1.1 million tonnes of CO_2_eq would be equivalent to 2.35 years' worth of total annual CO_2_ emissions of all sources in Kisumu at the current rate.

**Fig. 10:**
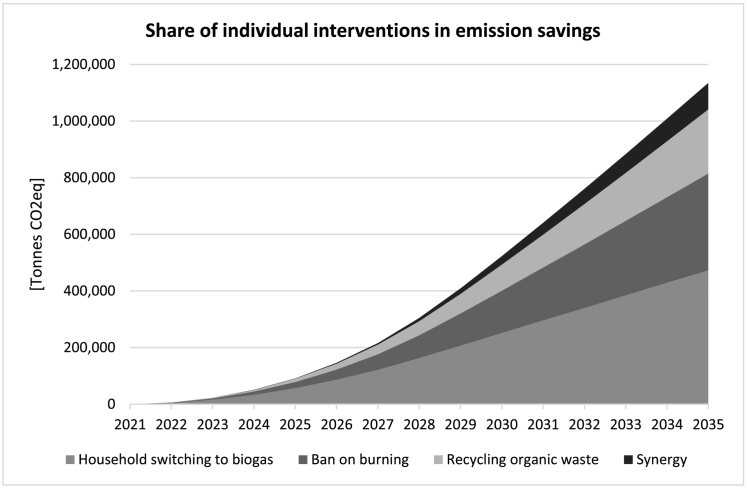
Share of individual interventions and synergy in total cumulative savings in GHG emissions.

Furthermore, it would be of interest to investigate the share of each individual intervention in the resulting cumulative savings in GHG emissions. This is visualised in Fig. 10 below. As can be seen, the largest contribution (42% of total in 2035) is derived as a result of the biogas produced replacing unclean fuels in the community's kitchens. On top of that there are significant savings (30% of total in 2035) thanks to the gradual enforcement of a ban on the open burning of waste, pointing to the crucial importance of enforcing such measure for reducing emissions. Next, we expect substantial savings (20% of total in 2035) in emissions associated with recycling part of the organic waste, diverting it away from landfill and into biogas production. Also interesting is the non-negligible portion of the savings (8% of total in 2035) that cannot be contributed to any individual intervention alone and is rather the synergistic outcome of simultaneous implementation of all interventions (the portion shown in black in Fig. 10). As we saw earlier (Fig. 9), the ban on burning policy alone significantly reduces emissions due to burning but at the same increases emissions due to waste decomposition, due to the higher levels of accumulated waste. Therefore, combining this intervention with the *Biogas* scenario which helps decrease the accumulation of food waste gives results that are superior to simply superimposing improvements from each separate intervention. Therefore, a ban on open burning together with the biogas production intervention helps maximise potential benefits.

### 3.5 PM_2.5_ concentrations

**Fig. 11:**
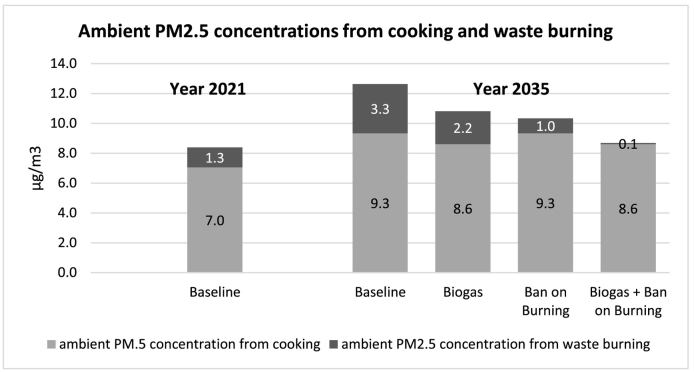
Changes in ambient PM_2.5_ concentrations from cooking and waste burning.

In this section, we look at results for changes in *ambient PM*_*2.5*_ *concentration from cooking and waste burning* as shown in Fig. 11. These results take into account PM_2.5_ emissions due to both the open burning of waste (dark grey) and household cooking (light grey). The totals are compared at present (*Year 2021*) versus at the end of our simulation period under our four scenarios (*Year 2035*). Concerning the demographics of cooking fuel types, the proportions of households using different fuel types are assumed to stay constant relative to each other (based on national statistics (KNBS, 2019)), except for the proportion of households using biogas which is endogenously and dynamically generated in the model. As this proportion goes up with expanding waste-to-biogas capacity, the proportion of households using other types of fuels decrease proportionately while staying constant relative to each other.

As seen above, at present the estimated average contribution of cooking to ambient PM_2.5_ concentration in Kisumu County is about 7.0 μg/m^3^ and the part attributed to waste burning is roughly 1.3 μg/m^3^, together adding roughly 8.3 μg/m^3^ to ambient PM_2.5_ concentration. Under the *Baseline* scenario, as a result of growth in population, this total is expected to rise by over 50% to 12.6 μg/m^3^. The *Biogas* scenario stands at a total of 10.8 μg/m^3^ by 2035, 14% lower than *Baseline*, with improvements coming from both sources (a transition to biogas for cooking as well as less waste being burnt). The *Ban on Burning* scenario brings a slightly more substantial reduction of 18% compared to *Baseline*, with all of this reduction naturally deriving from less waste burning (which only takes place in places other than landfill in this case). As expected, the highest reduction results from combining the two interventions, which brings total PM_2.5_ concentration from the two sources down to 8.7 μg/m^3^, over 30% lower than *Baseline*, and only 5% higher than the present level, despite the nearly 40% projected rise in population over the period.

Concerning household air pollution, the model projects an improvement of nearly 10%, from an average of 73.4 μg/m^3^ down to an average of 66.5 μg/m^3^ in indoor air concentration by 2035 as a result of a fraction of households (8.2%) being able to switch to biogas for cooking, as well as slightly improved ambient air pollution.

### 3.6 Health outcomes

**Fig. 12:**
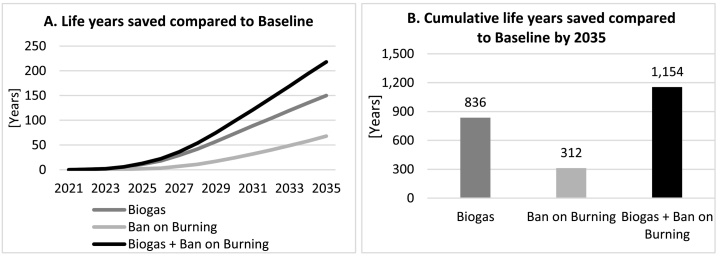
Life years saved compared to Baseline.

Fig. 12 shows results of the health impact assessment using life tables. Panel A presents total annual life years saved over the population of Kisumu, while panel B shows cumulative results by the end of the study period (2035). The highest impact is associated with the combined scenario, under which by 2035 we expect to see nearly 220 life years saved annually and a cumulative saving of over 1150 life years between 2020 and 2035. The *Biogas* intervention contributes approximately 70% of this estimated health benefit since it affects both indoor and outdoor air pollutant concentrations via reductions in the amount of waste burnt outdoors and the amount of unclean cooking fuels burnt indoors.

Given the time lags between changes in exposure and health outcomes, the estimated improvements are expected to grow substantially larger over time and would be greater than presented here if we were to extend the follow up period.

## 4 Discussion and conclusions

In this section, we will start with a summary of our findings and continue with a discussion of some of the implementation challenges of our proposed policies and conclude by briefly enumerating some of the limitations of this study and suggesting avenues for further research.

### 4.1 Summary of findings

Consistent with the qualitative forecasts of Awuor et al. (2019), Sibanda et al. (2017) and others, our results show that under business-as-usual, the state of waste accumulated in Kisumu's landfill is expected to worsen significantly, with the volume of waste reaching over 550,000 t by 2035, three times its current volume. Under the *Biogas* scenario however, which entails a gradual expansion of waste -to-biogas capacity up to 90,000 t per year by 2028, we can expect to see a reduction of about 7% in accumulated waste in landfill by 2035. As for scattered waste, under the *Biogas* scenario we can expect it to reach near zero by 2035, promising clean roads as a result of an assumed gradual expansion in the city's waste collection fleet. In addition, simulation suggests that, given our assumptions, by 2028 each year around 9 million m^3^ of biogas can be generated from biowaste, providing cooking fuel for 8–9% of total households in the Kisumu county. Under the *Biogas* scenario, total cumulative savings in emissions reach just over 700,000 t of CO_2_eq by 2035, two thirds of which come from the biogas replacing traditional fossil fuels for cooking.

On the other hand, with an assumed regulatory ban on the open burning of waste in landfill, waste is shown to accumulate more rapidly in landfill, as would be expected. However, aggregate GHG emissions decline notably, standing at about 35% lower than *Baseline* by 2035. Combining the two interventions, i.e., *Biogas* and *Ban on Burning,* gives over 1.1 million tonnes of cumulative savings by 2035. Out of this total, the largest contribution (42% in 2035) is derived as a result of the biogas produced replacing unclean fuels in the community's kitchens. This result is consistent with the findings of Møller et al. (2009) who report that indirect downstream emissions tend to be the most important factor in GHG accounting of waste-to-biogas initiatives.

With regards to air pollutant emissions and concentrations, combining the two interventions is expected to bring total PM_2.5_ emissions from the residential and waste sectors down by over 30% compared to *Baseline* by 2035; a level only 6% higher than present, despite the nearly 40% projected rise in population over the period. Furthermore, the model estimates a potential improvement of around 10% in indoor air PM_2.5_ concentrations by 2035 as a result of a fraction of households (8.2%) being able to switch to biogas for cooking, as well as improved ambient air quality. This mirrors the qualitative but empirical findings of Clemens et al. (2018), who report that 45%–91% of users in the Africa Biogas Partnership Program reported reduced eye problems and respiratory symptoms. Our health impact assessment suggests that these combined improvements in exposure can be expected to result in nearly 1150 cumulative life years saved by 2035, with an additional ~220 years or more added to those savings every year by that point.

### 4.2 Implementation challenges

In our modelling and analysis, we did not consider potential difficulties in the implementation of the interventions considered. Kemausuor et al. (2018) present a comprehensive review of barriers towards the uptake of biogas technology in Africa and maintain that, given the large initial investment costs, financing is at the heart of the barriers to extended uptake of biogas. Therefore, this study is part of a larger multi-partner effort to obtain funding for the described waste-to-biogas initiative from an international green climate fund. Other barriers identified by Twinomunuji et al. (2020) based on their case studies in Uganda and Ghana include varying enforcement of regulations, uncertainties around user experience with biogas including cooking preferences, and lack of in-country expertise. Furthermore, there are safety issues around operation of biogas installations having to do with the toxicity and the combustibility of biogas which can cause fires and explosions, although the associated risks are lower than chemical plants (Trávníček and Kotek, 2015).

In addition, transitioning towards our particular preferred scenario (*Scenario 2b. Biogas + Ban on Burning*) would require planning for and investing in the filling stations needed to make the product available to households, which poses an important technical and organisational challenge. It would also call for significant behavioural changes by households and other actors involved in the system. Firstly, households would need to sort their organic waste for collection. This has been identified as an ongoing challenge in Kisumu over several decades (Henry et al., 2006; M. Aurah, 2013; Sibanda et al., 2017; Awuor et al., 2019), although some household waste is sorted for composting and informal waste picking (Sibanda et al., 2017). Field studies suggest there is an interrelated set of barriers to efficient waste sorting at scale. One is that households and public spaces in the city lack segregated bins (Sibanda et al., 2017; Awuor et al., 2019). Where they are available, waste types are still often mixed either at the point of disposal, or when the bins are emptied and waste transported to the dumpsite (Sibanda et al., 2017; Awuor et al., 2019). Knowing this may undermine households' motivation to segregate waste. This might be further compounded by disagreement among stakeholders about who is responsible for the city's solid waste management, and a perceived mismatch between the government's expectations of the public and the public's willingness to participate in waste management (Schlueter, 2017).

Secondly, our combined scenario would require households to switch to and sustain the use of biogas as a cooking fuel. Despite the health, climate and economic advantages of switching from traditional to cleaner cooking fuels, studies in Kenya and other low- and middle-income settings indicate that such considerations do not necessarily drive sustained adoption (Jonušauskait, 2010; Rupf et al., 2015; Puzzolo et al., 2016; Chalise et al., 2018; Hamid and Blanchard, 2018; Thompson et al., 2018). Barriers identified among rural Kenyan communities to the sustained adoption of biogas included a lack of information and understanding about its use, benefits and cost-efficiency compared to traditional fuels (Ndereba, 2013; Hamid and Blanchard, 2018).

For both sorting waste and switching fuels, tools for designing and implementing behaviour change interventions may help achieve these transitions. Systems methods can also be used to understand the wider network of actions needed to support these changes (Gutberlet et al., 2017). Planned future work within the *Complex Urban Systems for Sustainability and Health (CUSSH)* programme (Belesova et al., 2018) will involve qualitative systems mapping of human behaviours involved in SWM in Kisumu to identify drivers of behaviour. From these, frameworks such as the Behaviour Change Wheel (Michie et al., 2011) may be applied to identify possible interventions which can be assessed for their suitability to the local context against criteria such as APEASE (Affordability, Practicality, Effectiveness and cost-effectiveness, Acceptability, Side-effects/safety, and Equity) (Michie et al., 2014).

Thirdly, there are likely to be several challenges towards implementing a ban on the open burning of waste in landfill. Since Kisumu's main landfill, Kachok dumpsite, is already overflowing, and since open burning is a key method used to reduce the volume of accumulated waste (Schlueter, 2017; Awuor et al., 2019), banning open burning, if unaccompanied by other interventions to reduce the inflow of waste to landfill and to keep waste levels down, can lead to more severe environmental problems due to waste overflow. Additionally, scavenging on dumpsites often involves the use of fire to recover recyclables such as tyre wire/tyre derived steel, and these practices are likely to continue unless alternative methods of recovering these materials are introduced. Furthermore, in open dumpsites spontaneous combustion can happen that is not humanly induced. Spontaneous combustion occurs when landfill waste is heated beyond ignition temperature as a result of exothermic reactions (Awuor et al., 2019).

### 4.3 Limitations

In building the model used in this study we have made a number of simplifying assumptions. For example, we have assumed that waste generation per household will stay constant over our simulation period of 15 years. However, Olang et al. (2018) have demonstrated that the amount of waste generated per household for Kisumu is dependent on factors such as household size and income. The model can be improved by incorporating these drivers based on any existing future projections for income and household size and by allowing waste generated per household to vary based on these.

Another key limitation of the model has to do with its choice of boundaries concerning the GHG accounting aspect, which includes only those components believed to be the most significant. The upstream-operating-downstream framework suggested by Gentil et al. (2009) includes several other components that, albeit less important in scale, represent useful potential additions to our model. These include leaked N_2_O and CH_4_ emissions from the biogas plant and digestate-related considerations (including fugitive and transport emissions and mineral fertiliser substitution savings).

Certain limitations are imposed on this study by the generally poor availability of data in the context of Kisumu. For instance, our estimation of PM_2.5_ emissions and particularly ambient concentrations resulting from them are subject to considerable uncertainty. While the GAINS model has been validated against ambient PM_2.5_ observations globally (Amann et al., 2020), we are not able to provide ground truthing of estimated PM_2.5_ concentrations in Kisumu due to the lack of ambient PM_2.5_ monitoring data there.

In addition, as explained in Appendix A (Section ii), the parameters we have used to estimate the *average household PM*_*2.5*_ *concentration* due to cooking are necessarily simplifications. Such estimates are obtained using a simplified method outlined in Appendix A (Section iv) and our focus is solely on the potential for biogas in reducing pollutant concentrations. The methodology for evaluating changes in indoor air PM_2.5_ concentration can be improved if empirical data on household air pollution for the context of Kisumu becomes available.

Moreover, with regards to capturing the health impacts of our scenarios, we have limited our analysis to the effects of particulate matter, while the risks associated with for instance contamination of Lake Victoria or flooding as a result of drainage systems being blocked by waste or the risks of vector-borne disease from breeding in water deposits in the waste are not considered, and therefore our reported health impact results are likely to be underestimates.

Lastly, concerning our *Biogas* scenario, while we have assumed the provision of substrate only from household food waste, a potentially promising alternative could involve an industrial symbiosis scenario where MSW is co-digested with waste from breweries operating in Kisumu. Under such scenario, the resulting biogas could be used not only for the required heat in the brewing process but also to produce electricity for the grid. There is an abundance of studies exploring the potential in co-digestion of brewery waste, although most studies appear to be in experimental and pilot stages (Tewelde et al., 2012; Murunga et al., 2016; Gunes et al., 2019).

Notwithstanding the above limitations, we maintain that, with respect to orders of magnitude and the relative performance of scenarios, our results are still valid and can be useful as a basis for policy planning over the medium term in the area of solid waste management in Kisumu. Findings can also provide informative background for policy planning in similar contexts.

In summary, the analysis presented in this paper demonstrates that a move towards recycling food waste to biogas for use in home cooking, along with a regulatory ban on the open burning of waste in landfill, can considerably mitigate the emission of GHGs and atmospheric pollutants in Kisumu. While helping the country towards achieving its emission reduction targets within the framework of the Paris Agreement, these measures also contribute to reducing the adverse impacts of waste and waste-related air pollution on public health. Having in mind the scarcity of health impact studies of environmental policy interventions in the context of Kisumu, as well as the rapid pace of change in this context and the opportunities this presents for sustainable development initiatives, we believe this study makes an important and timely contribution. The pioneering of Kisumu in reimagining its SWM system through measures such as those suggested in this paper can turn the County into a role model for others in Kenya, potentially providing a steppingstone towards a full revamping of SWM in the country, which can boost the positive impacts estimated in this study by orders of magnitude.

## CRediT authorship contribution statement

**K. Dianati:** Conceptualization, Methodology, Software, Formal analysis, Investigation, Writing – original draft, Visualization. **L. Schäfer:** Conceptualization, Validation, Investigation. **J. Milner:** Methodology, Software, Formal analysis, Writing – original draft, Writing – review & editing. **A. Gómez-Sanabria:** Methodology, Formal analysis, Writing – original draft, Writing – review & editing. **H. Gitau:** Investigation, Writing – original draft. **J. Hale:** Conceptualization, Writing – original draft, Writing – review & editing. **H. Langmaack:** Conceptualization, Investigation. **G. Kiesewetter:** Methodology, Software, Formal analysis, Writing – original draft. **K. Muindi:** Investigation, Writing – review & editing. **B. Mberu:** Supervision, Writing – review & editing. **N. Zimmermann:** Supervision, Writing – review & editing. **S. Michie:** Supervision, Writing – review & editing. **P. Wilkinson:** Conceptualization, Supervision, Writing – review & editing, Funding acquisition. **M. Davies:** Conceptualization, Supervision, Writing – review & editing, Funding acquisition.

## Declaration of competing interest

The authors declare that they have no known competing financial interests or personal relationships that could have appeared to influence the work reported in this paper.

## Figures and Tables

**Table 4:** Model coding explained, adapted from Dianati et al. (2019).

Code	Meaning
Lower-case variable	Endogenous variable (formulated based on other variables within the model). The dynamic behaviour of such variables is given by software simulation.
Upper-case variable	Constant. Such constants are either fixed parameters (black), or policy/scenario variables set by the user (green)
Variable with first word in upper case, rest in lower case	Exogenous (data) variable. Past behaviour of such variables is given by historical data. Variables stays constant for future simulation, unless otherwise specified.
Red variable	Key indicator.
Green variable	Policy/scenario variable, decided upon by the user.
Blue variable, in angle brackets	‘Shadow’ variable, copied from another section of the model.
Blue arrows	Causal relationships, from cause to effect. Each (endogenous) variable is formulated based on variables connected to it via incoming arrows.
Grey arrows	Initial condition setting.

**Fig. 13:**
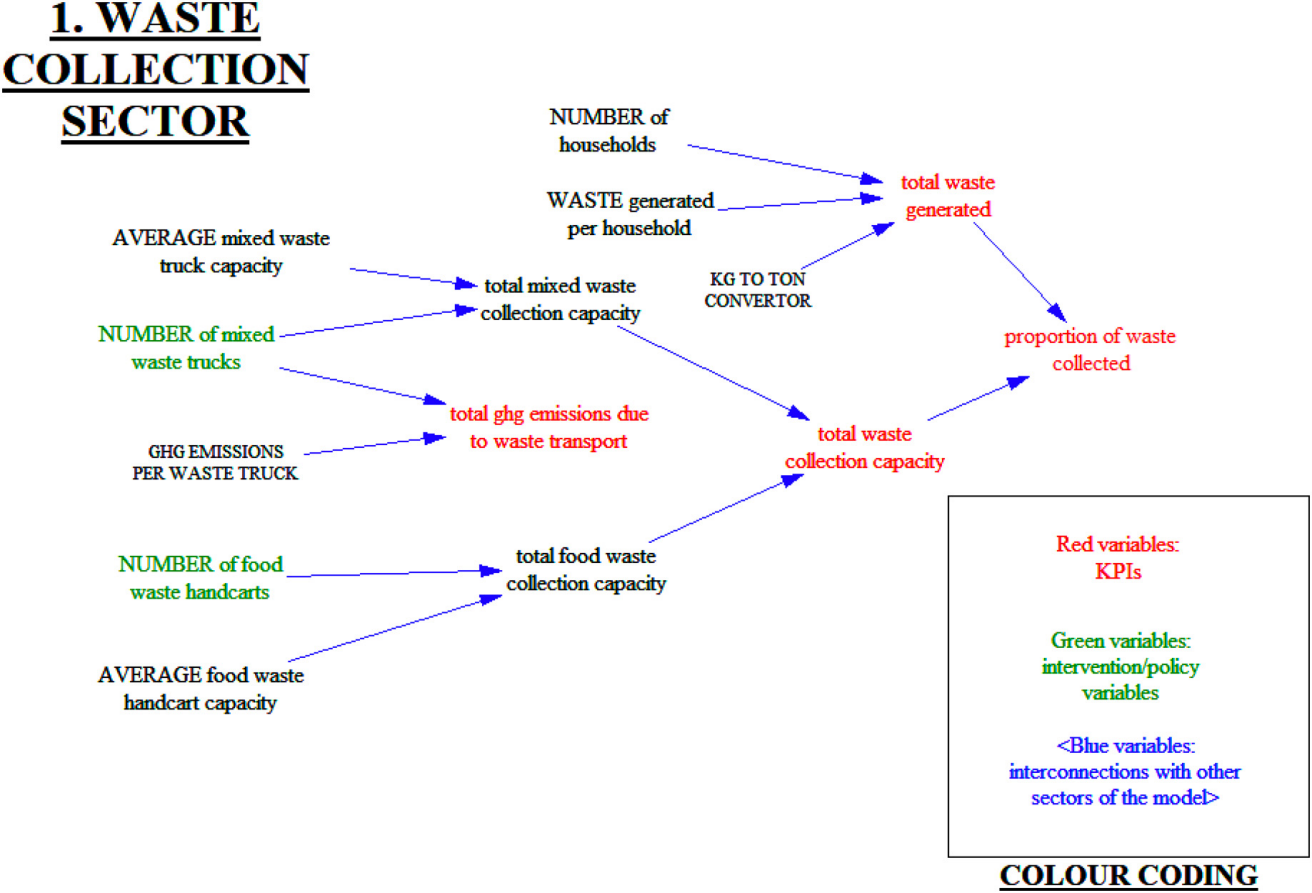
Sector one – waste collection.

**Fig. 14:**
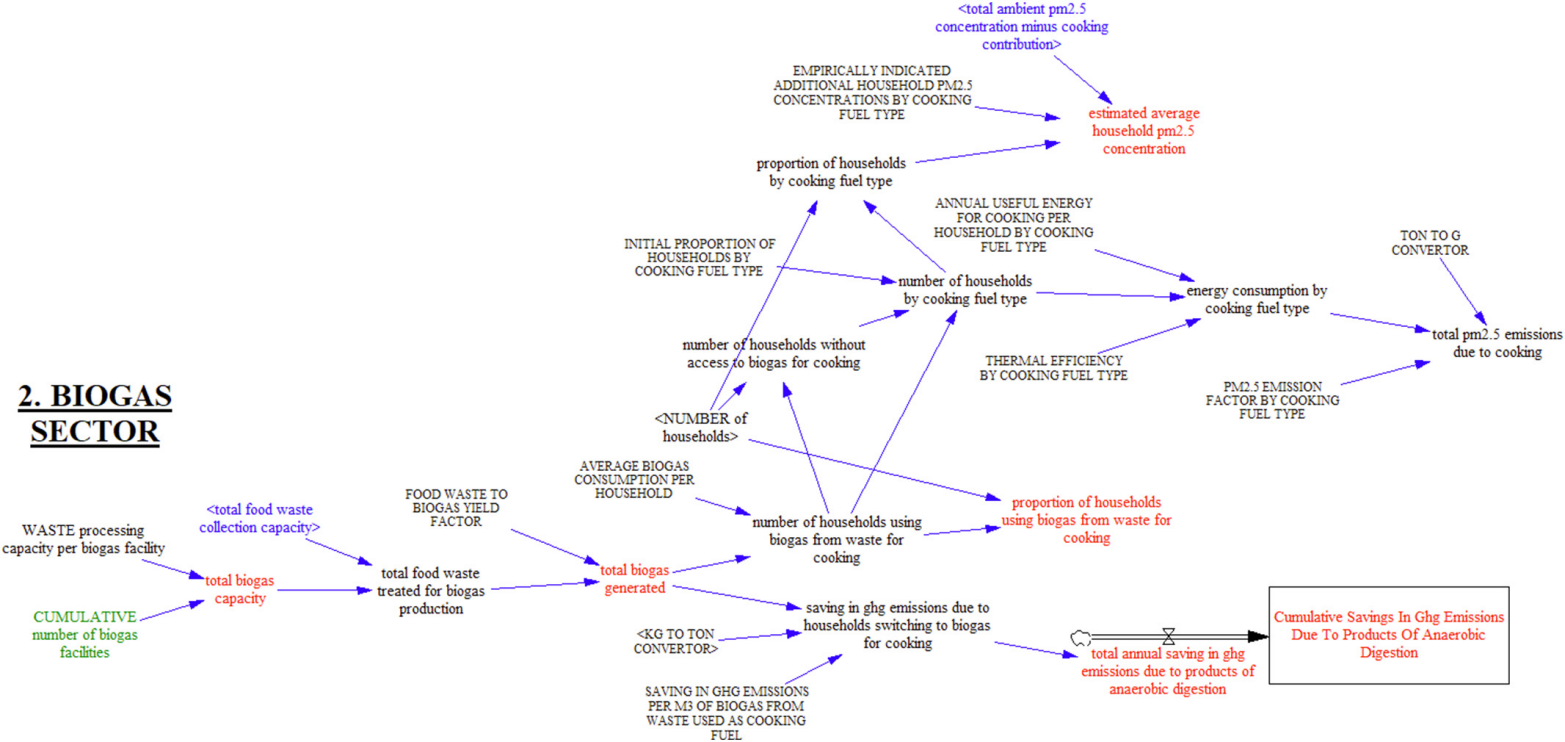
Biogas Sector.

**Fig. 15:**
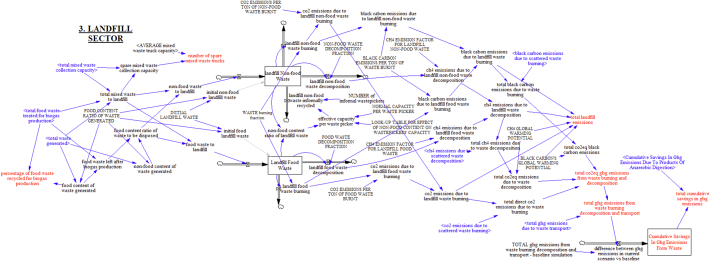
Landfill Sector.

**Fig. 16:**
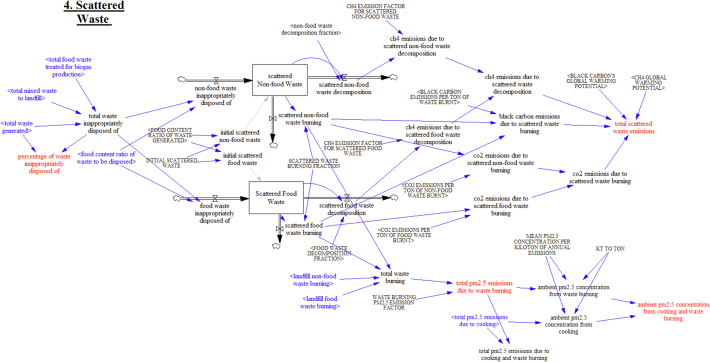
Scattered Waste Sector.

**Table:** 

Variable name	Note	Value	Unit	Source
Waste Collection Sector				
Waste generated per household	500 t per day divided by 301,000 households (representing 2017 population of 1,145,747 with a household size of 3.81, based on 2019 data)	606.3 kg per year or about 1.66 kg per day	kg/year or day	(Oyake-Ombis, 2017, p. 24)
Average mixed waste truck capacity	• Two trucks with a capacity of 10 t each, 6 trips each per day (2 × 10 × 6 = 120 t/day) • Two farm trucks with capacity 6 t and 4 t making 5 and 8 trips per day, respectively (6 × 5 + 4 × 8 = 62 t/day) • Skip loader with capacity 7 t, 6 trips daily on average (7 × 6 = 42 t/day)	120 + 62 + 42 = 224 t/day, combined capacity of 5 trucks 224/5 = 45 t/day average capacity of each truck	tonnes/day	Dumpsite Manager, Director Environment City and County Director Environment Conservation
GHG emissions per waste truck	Vehicles using 20 l per day of fuel Average Diesel truck CO2 emission is 2.63 kg/l. This makes 2.63 × 20 = 52.6 kg of CO2 emissions per day or 19.2 Tonne/Year.	19.2	tonnes/year	(DEFRA, 2007)

Biogas Sector				
Food waste to biogas yield factor		100	m^3^ per tonne	Veeken (2005) cited in (Müller, 2007, p. 26, Table 3)
Average biogas consumption per household	Equivalent to 125 kg LPG/household/year Nerini et al. (p.6). 1 kg LPG is equivalent to 2.10 m3 biogas (IRENA, 2016, Table 8, p18) ≥ 125*2.1 = 262.5 m3 per household per year	262.5	m^3^ per household per year	Nerini et al. (2017, p6), (IRENA, 2016, Table 8, p18)
Saving in GHG emissions per m^3^ of biogas from waste used as cooking fuel	…as opposed to equivalent energy from fossil fuels	4.93	kg emissions per m^3^ of biogas	(KNBS, 2019, p. 336) for types of cooking fuel used by households and (International Renewable Energy Agency (IRENA), 2016, pp. 18, 21) for converting those into potential savings
Initial proportion of households by cooking fuel type		Wood Open-Fire (OF): 31%, Wood Improved Cook Stove (ICS): 2%, Charcoal Trad (Traditional): 16%, Charcoal ICS: 30%, Kerosene: 13%, LPG: 5%, Electric: 1%, Biogas: 0%	%	(Carvalho et al., 2019, p. 173 Table 3)
Annual useful energy for cooking per household by cooking fuel type		Wood OF: 23,544, Wood ICS: 16,416, Charcoal Trad: 22,464, Charcoal ICS: 20,628, Kerosene: 10,476, LPG: 7236, Electric: 5879, Biogas: 7344	MJ/household/year	(Carvalho et al., 2019, p. 173 Table 3)
Thermal efficiency by cooking fuel type		Wood OF: 15%, Wood ICS: 24%, Charcoal Trad: 31%, Charcoal ICS: 38%, Kerosene: 42%, LPG: 54%, Electric: 71%, Biogas: 57%	%	(Carvalho et al., 2019, p. 173 Table 3)
PM_2.5_ emission factor by cooking fuel type		Wood OF: 14, Wood ICS: 8, Charcoal Trad: 9, Charcoal ICS: 18, Kerosene: 1, LPG: 1, Electric: 0, Biogas: 4	g PM_2.5_/MJ energy	(Carvalho et al., 2019, p. 172 Table 1)
GHG saving per kg of organic nitrogen replacing inorganic fertiliser		13.5	kg GHG per kg N	(Zhang et al., 2013)

Landfill Sector				
Waste decomposition fraction	Proportion of waste that is decomposed over a year?	10% for food waste and 1.67% for non-food waste	% per year	(Höglund Isaksson et al., 2016)
Waste burning fraction	Proportion of waste that is reduced over a year as a result of burning	23%	% per year	(Onyango and Kibwage, 2008)
CO_2_ emissions per tonne of food waste burnt		Zero	tonne of CO_2_ per tonne of waste	(IPCC, 2006, vol. 5 ch. 5)
CO_2_ emissions per tonne of non-food waste burnt		464.887	kg CO_2_ per tonne of waste burnt	See Section 2.3
CH_4_ emission factor for landfill food waste		20.27	kg CH_4_ per tonne of waste decomposed	See Section 2.3
CH_4_ emission factor for landfill non-food waste		17.70	kg CH_4_ per tonne of waste decomposed	See Section 2.3
Black carbon emissions per tonne of waste burnt		0.65	kg BC per tonne of waste burnt	(Akagi et al., 2011)
CH_4_ global warming potential		28	dmnl	(Meyer and Pachauri, 2014)
Black carbon's global warming potential		460	dmnl	(ICCT, 2009)
Normal capacity per waste picker		25	kg/day/person	Dumpsite manager
Initial landfill waste	Very rough estimate.	140,000	tonnes	Dumpsite manager
Food content ratio of waste generated		63%	%	(Gutberlet et al., 2017, p. 113)
Initial food content ratio of dumped waste		51.8%	%	(Aguko et al., 2018, p. 6)

Scattered Waste Sector				
Initial scattered waste		185,000	tonnes	See Scattered Waste Sector
CH_4_ emission factor for scattered food waste		10.13	kg CH_4_ per tonne of waste decomposed	See Section 2.3
CH_4_ emission factor for scattered non-food waste		8.85	kg CH_4_ per tonne of waste decomposed	See Section 2.3
Waste burning PM_2.5_ emission factor		8.74	kg PM_2.5_ per tonne of waste burnt	(Klimont et al., 2017, p. 8700)
Mean PM_2.5_ concentration per kt of annual emissions		2.05	(μg/m^3^)/(kt/year)	See Appendix A, Section (iv)

**Table:** 

Time	2021	2022	2023	2024	2025	2026	2027	2028	2029	2030	2031	2032	2033	2034	2035	Unit
1. Waste Collection Sector																
Waste generated per household	543.00															kg/household/year
Number of households	314,449	321,323	328,347	335,525	342,859	349,881	357,047	364,359	371,821	379,436	386,596	393,891	401,324	408,897	416,613	Households
Number of households growth rate	2.2%	2.2%	2.2%	2.2%	2.0%	2.0%	2.0%	2.0%	2.0%	1.9%	1.9%	1.9%	1.9%	1.9%		dmnl/year
Number of mixed waste trucks	5	5	6	6	7	7	8	8	9	9	10	10	11	11	12	dmnl
Average mixed waste truck capacity (tonnes per day)	45															tonne/day
Number of food waste handcarts (Scenarios 1 & 1b)	0	0	0	0	0	0	0	0	0	0	0	0	0	0	0	dmnl
Number of food waste handcarts (Scenarios 2 & 2b)	0	83	137	192	247	302	357	411	411	411	411	411	411	411	411	dmnl
Average food waste handcart capacity (kg per day)	600															kg/day

2. Biogas Sector																
Cumulative number of biogas facilities (Scenarios 1 & 1b)	0	0	0	0	0	0	0	0	0	0	0	0	0	0	0	dmnl
Cumulative number of biogas facilities (Scenarios 2 & 2b)	2	6	10	14	18	22	26	30	30	30	30	30	30	30	30	dmnl
Waste processing capacity per biogas facility	3000															tonne/year

3. Landfill Sector																
Number of informal waste-pickers	84	85	87	89	91	93	95	97	99	101	103	105	107	109	111	Persons
Waste burning fraction (Scenarios 1 & 2)	0.23	0.23	0.23	0.23	0.23	0.23	0.23	0.23	0.23	0.23	0.23	0.23	0.23	0.23	0.23	dmnl/year
Waste burning fraction (Scenarios 1b & 2b)	0.23	0.2	0.17	0.14	0.11	0.08	0.05	0.02	0	0	0	0	0	0	0	dmnl/year

## Appendix A Full model documentation

In this appendix, the formulation and parametrisation of the SD model is explained in detail. The model and the simulation runs are available as online supplementary material to this paper. The model is built in *Vensim*, a widely used SD simulation software package. The whole 120-plus-variable model is presented sector by sector, with visual snapshots to aid understanding. The following table elaborates the colour-coding and other information needed to interpret the diagrams.

As outlined earlier in the body of the paper, the model consists of four inter-connected sectors: (1) Waste Collection, (2) Biogas, (3) Landfill, and (4) Scattered Waste. In this section, the four sectors will be described in detail.

i) Waste Collection Sector

This sector, as depicted in Fig. 13, carries out simple accounting operations and involves no dynamic complexity (such as feedback loops, delays or accumulations). It is, however, useful in capturing the waste collection process within the case study in a visual and aggregate way.

Specifically, two different types of waste collection capacity are modelled: Firstly, the currently existing mixed waste collection trucks which transport the waste to landfill without any segregation of waste; and secondly, potential specialised waste collection capacity for separated food waste, whereby a number of waste handcarts would collect food waste from households (including in narrow alleyways of informal settlements where poor access prohibits the use of trucks) and take it to decentralised biogas production facilities, as modelled in the next sector. *Total mixed/food waste collection capacity* in Fig. 13 is the *number* of trucks/handcarts multiplied by the *average capacity* of the vehicles. The only currently existing capacity for waste collection consists of five mixed-waste trucks (with an average capacity of about 45 t per day^3^). *Total waste collection capacity*, which is the sum of all existing capacity plus any future added capacity, determines the *proportion of waste collected*, as a key indicator, on the right-hand side of the diagram. Currently, this *capacity* stands at about 225 t per day, which is about 43% of the total approximately 522 t per day of waste generated. Any remaining waste that is not collected is assumed to be inappropriately disposed of in open pits or scattered on roadsides and elsewhere, as modelled later in the Scattered Waste Sector. *Total GHG emissions due to waste transport*, which is accounted for in *total GHG emissions from waste* as seen later, is also calculated in this sector based on a constant level of *GHG emissions per waste truck* of around 52.6 kg of CO_2_ per day. This is based on an average consumption of around 20 l of diesel per day and an emission factor of 2.63 kg/l (DEFRA, 2007, p. 29).

ii) Biogas Sector

The second sector of the model includes another set of accounting equations for keeping track of the portion of waste that is – or rather could be, in the future – recycled into biogas and fertiliser. The sector is presented in two diagrams: Fig. 14 shows the structure where savings in GHG emissions are calculated.

Starting with Fig. 14, the *cumulative number of biogas facilities* is a ‘policy variable’, which means that it is a user-determined external input to the model that is based on our scenario assumptions (as described in Table 3). The resulting *total biogas capacity*, based on an average *waste processing capacity per biogas facility*, together with the concurrent restriction of *total food waste collection capacity* (imported from the previous sector), gives *total food waste treated for biogas production*. This determines *total biogas generated* based on a constant *food waste to biogas yield factor*, assumed equal to 100 m^3^ of biogas per tonne of food waste. This is based on the figure provided by Veeken (2005) cited in (Müller, 2007, p. 26) for plants of a similar scale and technology in India as those we envisage for commissioning in Kisumu. Subsequently, *total biogas generated* gives on the one hand the *number* and *proportion of households using biogas from waste for cooking*, which is determined based on a constant *average biogas consumption per household* of 262.5 m^3^ per household per year.^4^ On the other hand, *total biogas generated* can be used to calculate *saving in GHG emissions due to households switching to biogas for cooking* based on a constant *saving in GHG emissions per m*^*3*^ *of biogas from waste used as cooking fuel*, which is estimated at 4.93 kg/m^3^. This estimate is based on the current proportions of households using different types of cooking fuel in Kisumu (KNBS, 2019, p. 336) along with the GHG emission factors associated with those types of fuels (International Renewable Energy Agency (IRENA), 2016, pp. 18, 21).

This gives *total annual saving in GHG emissions due to products of anaerobic digestion*, which is accumulated in the stock of *cumulative savings in GHG emissions*. Stock variables, denoted inside a box, represent processes of accumulation in the real world, mathematically formulated via integration of the inflows minus the outflows, which are shown as valves flowing into or out of stocks.

The *number of households using biogas from waste for cooking*, as obtained above, is subsequently used to calculate *total PM*_*2.5*_ *emissions due to cooking* with regards to ambient air. The former is first subtracted from the total *number of households* to give *number of households without access to biogas for cooking*. This number is then multiplied by the *initial proportion of households by cooking fuel type*. This is a subscripted (vector) variable with eight elements representing different cooking fuel types, namely *wood OF* (open-fire), *wood ICS* (improved cookstove), *charcoal trad* (traditional), *charcoal ICS*, *kerosene*, *LPG*, *electric* and *biogas*. The proportion of households using each different fuel type is given by census data from the Kenyan National Bureau of Statistics (KNBS, 2019, p. 336). The product of this multiplication gives the *number of households by cooking fuel type* for the first seven elements of the vector. The value of the final element (*biogas*) is simply equal to the *number of households using biogas from waste for cooking*, which is driven by *total biogas generated*, as we saw above. This variable is then used to obtain total *energy consumption by cooking fuel type* via multiplying by the *annual useful energy for cooking per household by cooking fuel type* and dividing by the *thermal efficiency by cooking fuel type*. All these variables are similarly vector variables and the two sets of parameters for *thermal efficiency* and *useful energy* are based on Carvalho et al., 2019 (p. 173). The result is then multiplied by *PM*_*2.5*_ *emissions factor by cooking fuel type* (also from Carvalho et al., 2019, p. 172) to give the *total PM*_*2.5*_ *emissions due to cooking*. This *total* is added to emissions related to waste burning in the Scattered Waste Sector to obtain *ambient PM*_*2.5*_ *concentration from cooking and waste burning*.

The same piece of structure also gives a rough estimate for the *average household PM*_*2.5*_ *concentration* due to cooking. This estimate is based on empirical measurements reported in Muindi et al. (2016, p. 7 Table 3) on mean levels of indoor PM_2.5_ concentrations in households using different cooking fuel types. To the authors this appeared to be the best practical method to come up with this estimate. However, it must be noted that the method suffers from two major caveats: (1) Although the measurements are taken within the Kenyan context, they relate to a different setting, namely the Korogocho informal settlement within the peri-urban areas of Nairobi, and (2) The given measurements do not have the same level of granularity as the rest of the sector. Only three figures are given for households using (a) charcoal or wood (used for the first four elements in our vector), (b) kerosene and (c) LPG/electricity (used for the last three elements of our vector). Albeit very rough, we believe that using this method to obtain an estimate for the health impact of our different scenarios is superior to completely ignoring the effect of changes in indoor PM_2.5_ concentrations. Thus, the *estimated average household PM*_*2.5*_ *concentration* is obtained as the weighted average of the *empirically indicated household PM*_*2.5*_ *concentrations by cooking fuel type* where the weights are given by the *proportion of households by cooking fuel type*.

iii) Landfill Sector

All remaining waste which is collected but not treated for biogas production is sent to the landfill as much as the current waste collection capacity allows. The part which is not collected due to a lack of capacity is assumed to be disposed of inappropriately and scattered or dumped anywhere other than the main landfill, as modelled in the next sector. A fraction of the waste dumped in landfill or elsewhere is burnt to reduce volume (Klimont et al., 2017, p. 8700). Waste burning emits CO_2_ into the atmosphere, while the decomposition of organic waste emits CH_4_, both of which are greenhouse gases. The dumping of the waste in landfill and the resulting GHG emissions are captured via the structure introduced in this sector.

Fig. 15 shows the structure of this sector, which is a key part of the model as it captures the dynamics of the accumulation of waste as well as the potentially changing composition of the waste in landfill. Landfill waste is disaggregated into the two stocks of *landfill food* and *non-food waste*, as the two types have different profiles in terms of GHG emission potentials. The two stocks are similarly configured in terms of inflows and outflows.

The stocks are initialised according to estimates of total amount of waste currently existing in the city's main landfill, which is estimated by dumpsite management at around 140,000 t. This amount is split between the stocks of *food* and *non-food landfill waste* initially based on Aguko et al. (2018, p. 6), who estimate empirically that 51.8% of Kachok's waste content is organic.

*Total mixed waste to landfill* is the lesser value between *total mixed waste collection capacity* and the total waste that remains after recycling some of it for biogas (i.e. *total waste generated* minus *total food waste treated for biogas production*). These variables are imported from the Waste Collection Sector. A useful indicator calculated here is the *percentage of food waste recycled for biogas production*, i.e. *total food waste treated for biogas production* divided by *food content of waste generated*.

The split of the *total mixed waste to landfill* going into each of the two *food/non-food* stocks depends on a dynamic *food content ratio of waste to be disposed.* This *ratio* varies depending on how much of the food waste generated by households is used for biogas production. In order to calculate the *food content ratio of waste to be disposed* we first take out *total food waste being treated for biogas production* from *food content of waste generated* (which is 63% of *total waste generated* in the Kisumu context), obtaining the *food waste left after biogas production*. Dividing this by the sum of the same plus *non-food content of waste generated* (37% of *total waste generated*) gives the dynamic *food content ratio of waste to be disposed*. As we will see later in our scenarios, this ratio will naturally go down as we start to recycle a part of the food waste into biogas. Multiplying this ratio by *total mixed waste to landfill* gives total *food waste to landfill*, with the rest flowing into the *landfill non-food waste stock*.

As for the outflows, each stock has an outflow of waste burning. The fraction of waste burnt every year is assumed equal to 23% for all three stocks based on Onyango and Kibwage (2008) cited in Gutberlet et al., 2017 (p. 113). Note that in our *Ban on Burning* scenarios this *waste burning fraction* is linearly brought down to zero over eight years. Moreover, there are the outflows which represent waste decomposition. This is in accordance with the IPCC, 2006 (vol. 5 ch. 3) recommendation of using a first order-decay for calculating methane emissions. The *waste decomposition fraction* differs in each case, depending on the proportion of biodegradable matter for each type of waste. Höglund Isaksson et al. (2016) assumes an approximate average half-life of 10 years for fast-degrading organic waste like food waste, i.e. a rate of 10% per year, and 20 years, i.e. 5% per year, for slow-degrading waste such as paper, which constitutes one third of non-food waste in Kisumu. Therefore, *non-food waste decomposition fraction* is assumed equal to one third of 5% or 1.67% per year.

In the case of *landfill non-food waste*, an additional outflow of waste is captured which represents the *landfill non-food waste informally recycled* by scavengers who contribute towards recycling plastics, bottles, cans and metallic objects (Awuor et al., 2019). Based on our consultation with Kisumu county's waste officials, there are currently around 80 such informal workers, each collecting on average about 25 kg of waste every day. In the future, the number of informal waste-pickers is assumed to grow according to the projected population growth rate. Since the composition of the waste will change under our scenarios, we assume that the capacity of each waste-picker is a function of the non-food content ratio of the waste in landfill. We assume that this capacity stays at its current value of an estimated 25 kg/day per person under the current non-food content ratio. However, if the non-food content ratio goes down to zero or up to 100%, in conjunction with that, it is assumed that the waste-pickers' average capacity goes respectively down to zero or up to twice the current capacity (linearly, in both cases).

Next, we are going to calculate waste-related GHG emissions for landfill waste based on the outflows of waste burning and decomposition. This is done by multiplying the amount of food/non-food waste that is burnt or decomposed each year by the respective emission factors for CO_2_, CH_4_, and BC. The structure for making these calculations is shown in Fig. 15.

The method suggested in IPCC (2006, vol. 5 ch. 5) is applied here to estimate CO_2_ emissions from open waste burning. Emission factors are calculated for each type of waste based on the fossil carbon content. CO_2_ emissions from biogenic origin are not included in the estimates as stated in the IPCC (2006, vol. 5 ch. 5). This means that CO_2_ emissions from open burning of food, paper and wood waste are set to zero. The implied CO_2_ emission factor for *landfill non-food waste burning* for the particular waste composition is Kisumu is assumed to be 464.89 kg CO_2_ per tonne of waste burnt (see Section 2.3 for details on these calculations).

The other important contributor to global warming which results from the incomplete combustion of waste is black carbon (BC). BC is a carbonaceous aerosol (Klimont et al., 2017) with a global warming potential^5^ (GWP) of 460 based on a 100-year time horizon (IPCC, 2007). The emission factor used here to estimate BC emissions is 0.65 kg BC per tonne of waste burnt (Akagi et al., 2011, p. 4047).

CH_4_ emissions are calculated and summed up using an emission factor of 17.70 kg per tonne of waste decomposed for *landfill non-food waste* and 20.27 kg for *landfill food waste* (see Section 2.3). Total CH_4_ emissions from the two stocks is then summed up with *CH*_*4*_ *emissions due to scattered waste decomposition* as obtained in the next sector (Scattered Waste Sector) and the sum total converted to CO_2_ equivalent using methane's GWP of 28 (Meyer and Pachauri, 2014). Summing up the resulting *total CO*_*2*_*eq methane emissions due to waste decomposition* with *total direct CO*_*2*_ *emissions due to waste burning* and *total CO*_*2*_*eq black carbon emissions* (both including emissions for landfill as well as scattered waste imported from the Scattered Waste Sector gives *total GHG emissions from waste burning and decomposition*. Finally, summing this up with *total GHG emissions due to waste transport*, imported from the Waste Collection Sector, gives *total GHG emissions from waste burning, decomposition and transport*. Using the additional stock variable of *cumulative savings in GHG emissions from waste*, we accumulate the *difference between GHG emissions in current scenario* versus *baseline*. Adding the value of this stock with *cumulative savings in GHG emissions due to products of anaerobic digestion*, imported from the Biogas Sector, gives our final key indicator of interest for each scenario, i.e. *total cumulative savings in GHG emissions*.

iv) Scattered Waste Sector

The final sector captures the accumulation of waste anywhere other than in landfill, such as on the roadside or in open pits, along with the resulting GHG and PM_2.5_ emissions. This sector (Fig. 16) is constructed very similarly to the previous one, in the sense that scattered waste is conceptually divided between the two stocks of *scattered food* and *non-food waste*, with similarly configured inflows and outflows.

What remains of *total waste generated* after subtracting *total food waste treated for biogas production* (Biogas Sector) and *total mixed waste to landfill* (Landfill Sector) constitutes *total waste inappropriately disposed of*, which finds its way into one of the two stocks, depending on the dynamic *food content ratio of waste to be disposed* as calculated in the Landfill Sector. The stock of *scattered waste* is initialised in relation to the stocks of *landfill waste*. Based on an initial *total waste generated* of around 522 t per day in 2021 and an initial *total mixed waste collection capacity* of around 225 t per day, it is estimated that initially around 225/522 = 43% of the waste is being collected and the remaining 57% is inappropriately disposed of in places other than the landfill. Therefore, it is considered a fair assumption that the ratio of *initial scattered waste* to *initial landfill waste* should also be close to 57/43. Given the rough estimate of 140,000 t for *initial landfill waste*, we reach an estimate of 185,000 t for *initial scattered waste*. As before, each stock has two outflows of *decomposition* and *burning* with the same *fractions* previously used for *landfill waste*.

Resulting GHG emissions are calculated in the same way and with emissions factors equal to those of the respective types of *landfill waste*, except for CH_4_ emissions which are calculated using different emission factors. This is because in the case of *scattered waste* there is assumed to be a lower level of compacting and therefore weaker anaerobic conditions resulting in lower CH_4_ emission factors for *scattered waste* as compared to *landfill waste*. CH_4_ emission factors for *scattered food* and *non-food waste* are assumed to be 10.13 and 8.85 kg CH_4_ per tonne of waste decomposed respectively (see Section 2.3). *Total scattered waste emissions* is calculated by summing up direct *CO*_*2*_ *emissions due to waste burning,* CO_2_ equivalent *black carbon emissions due to scattered waste burning*, and CO_2_ equivalent *CH*_*4*_ *emissions due to scattered waste decomposition*.

Additionally, in this sector *total PM*_*2.5*_ *emissions due to waste burning* is calculated by summing up the burning rates of different types of waste (*food/non-food; landfill/scattered*) and multiplying by *waste burning PM*_*2.5*_ *emission factor*. This factor is set to 8.74 kg PM_2.5_ per tonne of waste burnt based on (Klimont et al., 2017, p. 8700) for all different types of waste as an approximation. *Total PM*_*2.5*_ *emissions due to cooking* is imported from the Biogas Sector.

Each of the two *emissions* figures is converted into an implied figure for *concentration* via multiplying by a constant *mean pm*_*2.5*_ *concentration per kt of annual emissions* of 2.05 (μg/m^3^)/(kt/year). This coefficient is derived from the global atmospheric calculations in the GAINS integrated assessment model (Amann et al., 2020), which themselves rely on a linearized version of full atmospheric chemistry transport model simulations. Since Kenya is a source region in GAINS, the model contains atmospheric transfer coefficients from all source pollutants for PM_2.5_ from Kenya to a 0.1° receptor grid. Here we use the coefficient for primary PM_2.5_ emissions from urban low-level sources in Kenya in conjunction with the share of Kisumu's population in total Kenyan urban population to first calculate the response of ambient PM_2.5_ concentrations at 0.1° resolution to primary PM emissions from Kisumu, and then take a population-weighted average across all grid cells in the city to derive an integrated coefficient from Kisumu to itself. Thereby, we take two simplifying assumptions: 1) that only primary PM emissions play a role and local secondary particle formation can be neglected, and 2) that the contribution from such sources in other Kenyan cities to ambient PM in Kisumu is low. Assumption 1 may lead to a small underestimation of the coefficient, while assumption 2 may lead to a small overestimation. Given the large uncertainties in emissions, these simplifications seem justified.

The sum of the two implied concentrations gives an estimate for *ambient PM*_*2.5*_ *concentration from cooking and waste burning*. Changes in the resulting concentrations have important health implications, as reported earlier in the Results chapter.

## Appendix D Supplementary data

Ban on Burning Scenario. Image 1

Baseline simulation. Image 2

Biogas + Ban on Burning Scenario. Image 3

Biogas Scenario. Image 4

Data file required to run the model in Vensim. Image 5

Model (Vensim file). Image 6

## Footnotes

1 Estimates for the amount of waste generated in Kisumu vary widely, from 200 up to 500 t/day (Sibanda et al., 2017). The wide range of these estimates is due to the lack of systems to formally weigh generated waste (e.g. via a weighbridge at dumpsites), leaving the Kisumu Department of Environment to rely on rough per capita methods for estimating waste volumes. Here, we use the Oyake-Ombis (2017) figure which covers the entire Kisumu County.

2 Here, we assume that all waste burning in the dumpsite is humanly induced. This is, however, not necessarily the case as spontaneous combustion without human intervention also occurs.
